# Vitamin D deficiency in non-scarring and scarring alopecias: a systematic review and meta-analysis

**DOI:** 10.3389/fnut.2024.1479337

**Published:** 2024-10-02

**Authors:** Tanat Yongpisarn, Kasama Tejapira, Kunlawat Thadanipon, Poonkiat Suchonwanit

**Affiliations:** ^1^Division of Dermatology, Department of Medicine, Faculty of Medicine Ramathibodi Hospital, Mahidol University, Bangkok, Thailand; ^2^Department of Clinical Epidemiology and Biostatistics, Faculty of Medicine Ramathibodi Hospital, Mahidol University, Bangkok, Thailand

**Keywords:** vitamin D insufficiency, vitamin D level, hair loss, non-cicatricial alopecia, cicatricial alopecia

## Abstract

**Background:**

Numerous studies have linked vitamin D deficiency (VDD) to the pathogenesis of various alopecia disorders.

**Objective:**

This study aimed to investigate whether patients with alopecia are more likely to have VDD or lower vitamin D levels than controls, and the prevalence of VDD among patients with certain alopecia disorders.

**Methods:**

Electronic searches were conducted using PubMed, Embase, Scopus, and Cochrane Library databases from the dates of their inception until September 2024. Studies that reported data allowing for the calculation of odds ratios, mean differences, or correlation coefficients related to vitamin D levels and alopecia were included, while studies without a confirmed diagnosis of alopecia or those involving patients taking vitamin D supplements were excluded.

**Results:**

It was found that 51.94% of patients with alopecia areata (AA), 50.38% of patients with female pattern hair loss (FPHL), 47.38% of patients with male androgenic alopecia (MAGA), 53.51% of patients with telogen effluvium (TE), and 38.85% of patients with primary scarring alopecia had VDD. Compared to controls, AA patients had a pooled odds ratio (OR) of VDD of 2.84 (95% confidence interval: 1.89–4.26, *I^2^* = 84.29%, *p* < 0.01) and a pooled unstandardized mean difference (UMD) of vitamin D levels of −8.20 (−10.28 – −6.12, *I^2^* = 74.25%, *p* < 0.01) ng/mL. For FPHL patients, a pooled OR of VDD of 5.24 (1.50–18.33, *I^2^* = 81.65%, *p* < 0.01) and a pooled UMD of vitamin D levels of −15.67 (−24.55 – −6.79, *I^2^* = 91.60%, *p* < 0.01) ng/mL were found. However, for MAGA, a pooled VDD OR of 4.42 (0.53–36.61, *I^2^* = 88.40%, *p* < 0.01), and a pooled UMD of vitamin D levels of −2.19 ng/mL (−4.07 – −0.31 ng/mL, *I^2^* = 7.64%, *p* = 0.37) were found. For TE patients, pooled UMD of vitamin D levels of −5.71 (−10.10 – −1.32) ng/mL were found.

**Conclusion:**

People with alopecia frequently have VDD; however, only in patients with AA or FPHL was the association of VDD and decreased vitamin D levels statistically significant compared to control. The findings indicate screening for vitamin D could benefit patients with AA or FPHL, potentially addressing vitamin D deficiency. Further study on vitamin D supplementation as a treatment for alopecia is recommended.

## 1 Introduction

Vitamin D is a lipophilic hormone widely recognized as essential for bone development and calcium homeostasis, and it exerts its effect through the nuclear hormone receptor vitamin D receptor (VDR). Vitamin D is produced in the skin when exposed to sunlight and can also be obtained through diet. The liver synthesizes the primary form of vitamin D, 25-hydroxyvitamin D or 25(OH)D_3_, which is then activated in the kidneys by 1a-hydroxylase to produce its biologically active form, 1,25-dihydroxyvitamin D or 1,25(OH)_2_D_3_ (1, 2).

VDR is expressed by T and B lymphocytes, dendritic cells, and macrophages, and 1,25(OH)_2_D_3_ is known to modulate both innate and adaptive immune systems (3). Vitamin D deficiency (VDD) is believed to be an environmental trigger for the onset of autoimmunity, and many studies have found a link between VDD and autoimmune diseases (2–4). The VDR plays a crucial role in hair follicle cycling by regulating hair growth phases, particularly the transition from the anagen phase to the catagen phase (5). Additionally, VDR modulates the immune response in alopecia by interacting with key immune cells, such as T and B lymphocytes, macrophages, and dendritic cells, which are involved in the pathogenesis of autoimmune disorders (6). Non-immune-mediated alopecias (e.g., androgenetic alopecia [AGA] and telogen effluvium [TE]) and immune-mediated hair disorders (e.g., alopecia areata [AA] and primary cicatricial alopecia [PCA] such as frontal fibrosing alopecia [FFA], central centrifugal cicatricial alopecia [CCCA], and lichen planopilaris [LPP]) may therefore be associated with VDD (7, 8).

Alopecia can be classified into non-scarring and scarring types. Non-scarring alopecias are characterized by hair loss without permanent damage to hair follicles, while scarring alopecias result in permanent destruction of hair follicles due to inflammation and fibrosis (9). Alopecia is a well-known clinical sign of hereditary vitamin D resistant rickets (HVDRR), a rare disease caused by mutations in VDR. Growing evidence indicates that VDR plays a crucial role in normal hair cycling (10, 11). However, the relationship between blood vitamin D levels, tissue vitamin D concentrations, and VDR function remains to be researched since the impact of vitamin D levels on VDR function is complex and may depend on receptor sensitivity, co-regulators, and target gene expression (12). Although it is unknown whether or not deficient vitamin D levels in the blood would lead to deficient vitamin D in the tissue and whether or not this would lead to VDR dysfunction, numerous studies have linked VDD to the pathogenesis of various alopecia disorders, with a focus on AA, male androgenetic alopecia (MAGA), and female pattern hair loss (FPHL). While these studies demonstrate varying degrees of association between VDD and alopecia, there is still debate over the causality and consistency of findings (13–16). We aimed to conduct a systematic review and meta-analysis to determine the prevalence of VDD among various alopecia disorders, namely AA, AGA, TE, and PCA, the odds of VDD and differences in vitamin D levels of patients with various alopecia disorders compared to controls, and whether vitamin D levels are correlated with the severity of alopecia.

## 2 Materials and methods

### 2.1 Study design

The protocol was registered in PROSPERO (International Prospective Register of Systematic Reviews; CRD42023387901, https://www.crd.york.ac.uk/prospero/display_record.php?RecordID=387901). The systematic review followed the Preferred Reporting Items for Systematic Reviews and Meta-analyses guidelines (Supplementary Document 1) (17). Electronic searches were conducted from the database’s inception to September 2024 using the PubMed, Embase, Scopus, and Cochrane Library databases. The search strategy was designed to retrieve all studies on vitamin D, VDD, and alopecia using keywords and a controlled vocabulary. There were no restrictions on the language or publication period of the searches. Conference abstracts were excluded. The search included a combination of terms: ‘vitamin D,’ ‘vitamin D deficiency’, ‘alopecia,’ and ‘hair loss;’ with synonyms, related terms, and subject headings also used. Boolean operators (AND, OR) were used to combine terms. Grey literature and unpublished data were not considered. Supplementary Table S1 provides details about the search strategy.

### 2.2 Study selection

Two reviewers (TY and KaT) independently evaluated each article at both the full-text and title/abstract levels. Disagreements between the two reviewers regarding the studies’ eligibility were resolved via discussion with a third reviewer (PS). We included randomized controlled trials, cohort studies, and case–control studies that provided data for the calculation of the odds ratio (OR) of VDD, the mean difference in vitamin D level between cases and controls, the prevalence of VDD among patients with certain alopecia disorders, or the correlation coefficient (CC) between vitamin D level and alopecia severity. Studies involving patients without a confirmed diagnosis of alopecia or those taking vitamin D supplements were excluded to ensure consistency in the data. Our review questions in the format of PICO (population, intervention, comparator, and outcomes) are provided in Supplementary Table S2. We excluded studies that did not provide a specific type diagnosis of alopecia as well as those that included patients taking vitamin D supplements.

### 2.3 Data extraction

Data were extracted from the included studies using a standardized form. The following data were collected: bibliographic data (authors, year of publication), study characteristics (type of study, single or multicenter, study duration, country), alopecia group characteristics (number, age, gender, Fitzpatrick skin type (FST), ethnicity, body mass index (BMI), comorbidity, smoking status, alcohol consumption status, diet, sun-exposure, sunscreen usage, disease duration, disease severity score or grading (e.g., mean Severity of Alopecia Tool (SALT) score), alopecia pattern (e.g., % single patch, % multiple patches, % patchy type, % ophiasis type, % alopecia totalis (AT), % alopecia universalis (AU), % body site involvement), family history of alopecia, % nail involvement, % first episode, % recurrent episode, % stable/gradual disease, % active disease, treatment information, whether the diagnosis and severity assessment were done by dermatologist), control group characteristics (number, age, gender, FST, ethnicity, BMI, comorbidity, smoking status, alcohol consumption status, diet, sun exposure, sunscreen usage, whether controls were matched for any relevant factors), vitamin D results data (frequency data of VDD, vitamin D level, correlation coefficient, and relevant descriptive data), vitamin D measurement (VDD definitions, if any conversion was done, measurement methods), and other (exclusion criteria, if any conversion or data retrieval was done). Vitamin D levels were reported in various units across studies (ng/mL and nmol/L). To maintain consistency, all vitamin D values were converted to ng/mL using standard conversion methods. This ensured comparability of results across different vitamin D assays used in the included studies.

Because 1,25(OH)_2_D_3_ has a half-life of less than 4 h and the levels may remain normal in VDD, whereas 25(OH)D has a half-life of approximately 2 weeks, 25(OH)D is a stable indicator of vitamin D status and is routinely measured (18). In this review, the vitamin D level is therefore referred to as the 25(OH)D level.

Corresponding investigators were contacted via email if there was missing data. Two independent reviewers (TY and KaT) extracted data, and discrepancies were resolved with the assistance of a third reviewer (PS).

### 2.4 Quality assessment

TY and KaT independently assessed the quality of descriptive and case–control studies using the adapted version of the Newcastle-Ottawa Scale (NOS) (19). The NOS is a scoring tool comprised of seven items with nine scores that assess how well the investigators selected their participants (score ranges from 0 to 4), the comparability of their results (score ranges from 0 to 2), and the applicability of the outcomes (score ranges from 0 to 3). The higher the score, the higher the study’s quality and the lower the likelihood of bias. Therefore, we classified studies as having high quality if they received a total score of 7 or more, fair quality if they received a score of 4–6, and low quality if they received a score of less than 4. Any discrepancies between reviewers regarding the risk of bias in specific studies were resolved through discussion with a third reviewer (PS). The modified NOS used in our review is shown in Supplementary Table S3.

### 2.5 Statistical analysis

A meta-analysis was performed to pool the effect sizes, including the OR of a certain alopecia disorder and VDD, the unstandardized mean difference (UMD) of serum vitamin D level between subjects with a certain alopecia disorder and those without, the CC between vitamin D level and the SALT score. Additionally, the “metaprop” command with the Freeman-Tukey double arcsine transformation to stabilize the variances was used in Stata to pool the prevalence of VDD among various alopecia disorders (20). Each alopecia disorder was analyzed separately, and data from adult and pediatric populations was pooled independently. However, as a limited number of studies of scarring alopecia were expected, primary scarring alopecia diseases were planned to be analyzed based on their etiology, such as lymphocytic, neutrophilic, and mixed cell scarring alopecia (9).

Heterogeneity was assessed and considered present if a Cochrane Q test *p*-value was <0.1 or Higgins *I^2^* ≥ 25% (21). Subgroup analyses were further performed to explore potential sources of heterogeneity. Effect sizes were pooled using the DerSimonian and Laird method if they were heterogeneous; otherwise, the inverse-variance method was used (21). The sources of heterogeneity were explored by fitting each covariate (e.g., age, female gender, disease duration, BMI, active disease, relapse disease, severe AA, and SALT score) at a time in a meta-regression model. If the τ2 was decreased by ≥50% or statistically significant *β* was revealed, a subgroup analysis was performed based on that covariate (22). In addition, certain pre-planned subgroup analyses (country of research origin, age group, and alopecia severity) were also performed. Severe AA is defined as AT, AU, or extensive AA, and an AA cohort is considered severe AA if it has a mean SALT score ≥ 50% or ≥ 20% severe AA. We also conducted sensitivity analyses including only studies with high quality according to the NOS (studies receiving a total NOS score of 7 or more).

To evaluate publication bias, Deeks funnel plots of the primary outcomes were generated. The Egger linear regression test was applied when a funnel plot suggested possible asymmetry (23). If Egger’s test for a regression intercept gave a *p*-value <0.05, a contour-enhanced funnel plot was used to determine the cause of the asymmetry (23). STATA 16.0 (StataCorp LLC, College Station, TX, United States) was used for all statistical analysis.

## 3 Results

### 3.1 Study characteristics

**Figure 1:**
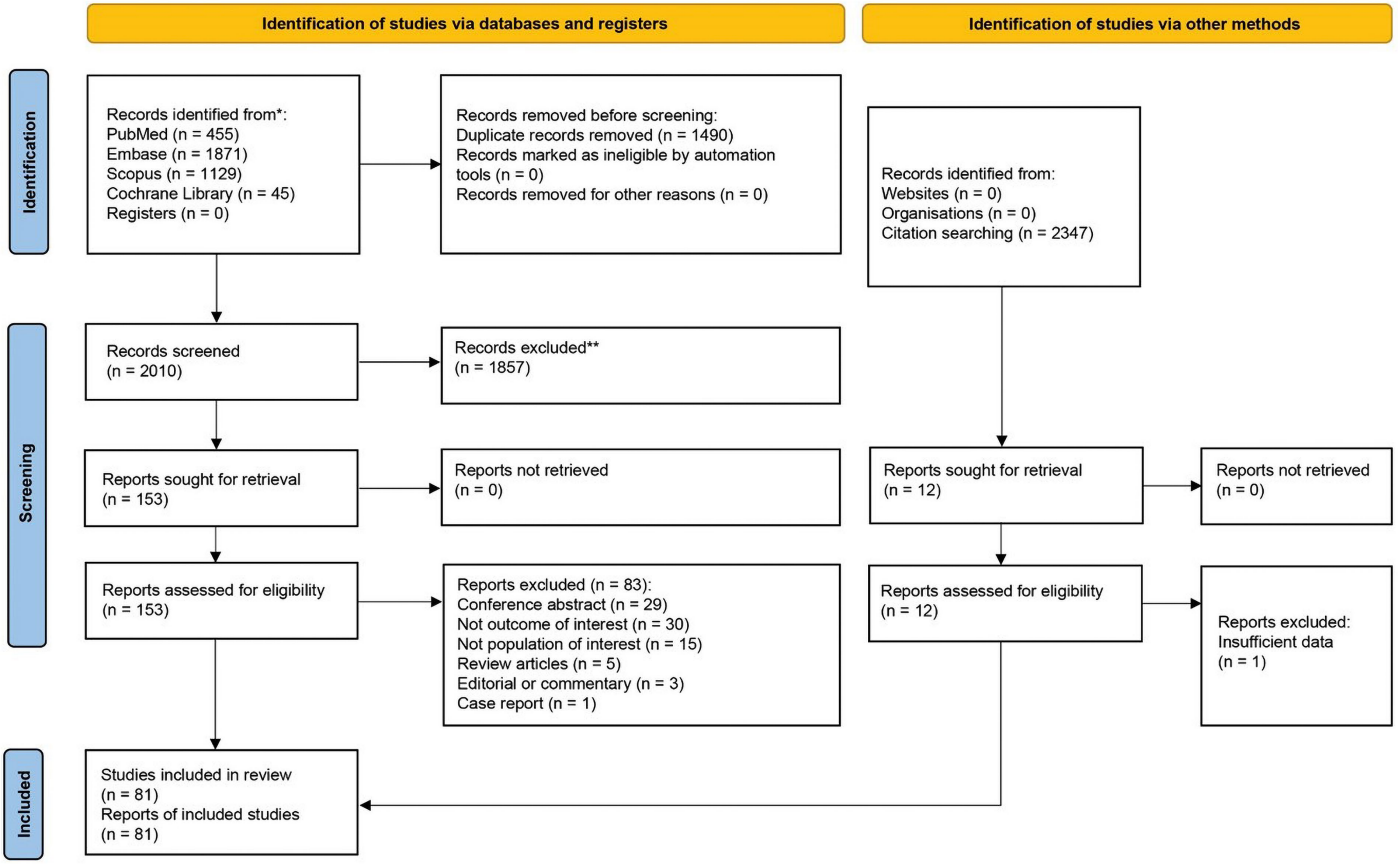
Preferred reporting items for systematic reviews and meta-analyses (PRISMA) flow diagram of search strategy and included studies.

After removing duplicates, 2010 references were screened by title/abstract. At the full-text stage, 153 full articles met our predefined selection criteria and were sought. We further excluded 83 references for the following reasons: conference abstracts (*n* = 29), not outcome of interest (i.e., no documented vitamin D deficiency prevalence or vitamin D level of the patients, *n* = 30), not population of interest (i.e., non-specific alopecia diagnosis and specialized hair loss disorders [e.g., drug-induced alopecia, hair loss associated congenital disorders, *n* = 15), review articles (*n* = 5), editorial or commentary (*n* = 3), and case report (*n* = 1)] (Figure 1). Twelve additional studies were identified by manually searching reference lists of included studies and relevant review articles, and 1 study was removed due to insufficient data. The review included 81 studies (79 studies were included in the quantitative analysis; 2 studies of pediatric non-scarring alopecia (24, 25) were excluded from the quantitative analysis), enrolling a total of 15,339 patients with alopecia [8,639 AA patients (13–16, 26–69), 2,943 AGA patients (14, 16, 27, 31, 34, 37, 70–86), 3,048 TE patients (27, 31, 34, 37, 78, 81, 84, 87–96), 489 LPP patients (34, 37, 97, 98), 107 FFA patients (37, 70), and 113 CCCA patients (34, 37, 99, 100)] between 2011 and 2024, were included in the review. Characteristic features of the included studies are provided in Tables 1–4 and Supplementary Table S4.

**Table 1:** Characteristics of the included studies involving patients with alopecia areata.

Author, year	Country	Study design	Group (case/control)	Mean age (SD)	Female (%)	Severity of alopecia
Hasanbeyzade and Tunca (2024) (68)	Turkey	Case–control	Patients with AA (41)	26.80 (7.00)	6 (14.60)	AT 10 (24.4%), AU 9 (22.0%), patchy AA 11 (26.8%), diffuse AA 11 (26.8%)
			Age- and sex-matched healthy controls (41)	26.90 (6.90)	5 (12.20)	–
AbdElneam et al. (2024) (63)	Saudi Arabia	Case–control	Patients with AA (82)	25 (3.90)	40 (48.80)	Localized patchy 38 (46.4%), Multiple patchy 31 (37.8%), Ophiasis 13 (15.8%)
			Age-matched healthy controls (81)	23.8 (2.80)	45 (55.60)	–
Saleem et al. (2023) (69)	Pakistan	Case–control	Patients with AA (45)	22.94 (7.92)	18 (40)	SALT class; S1 = 7 (15.6%), S2 = 10 (22.2%), S3 = 18 (40%), S4 = 3 (6.7%), S5 = 7 (15.6%), mean SALT score 56.3%^§^
			Age- and sex-matched healthy controls (45)	23.84 (8.46)	18 (40)	–
Hamidpour et al. (2023) (67)	Iran	Descriptive	Patients with AA (402)	27.20 (13.40)	192 (47.80)	Median SALT score 68 (IQR 40–100)
Gupta et al. (2023) (66)	India	Case–control	Patients with AA (25)	27.64 (9.83)	8 (32)	SALT class; S1 = 20 (80%), S2 = 2 (8%), S3-5 = 3 (12%)
			Age- and sex-matched healthy controls (25)	28.56 (7.95)	8 (32)	–
Fahim et al. (2023) (65)	Pakistan	Descriptive	Patients with AA (100)	30.50 (8.40)	58 (58)	Mean SALT score 20.7 ± 5.4
Alsenaid et al. (2023) (64)	Saudi Arabia	Case–control	Patients with AA (59)	27.10 (9.10)	6 (10.20)	Moderate 6 (10.2%), severe 9 (15.3%)
			Age-matched healthy controls (60)	27.4 (10.30)	9 (15)	–
Das (2022) (26)	India	Case–control	Patients with AA (50)	25.07 (7.40)	18 (36)	SALT class; S1 = 35 (70%), S2 = 10 (20%), S3 = 5 (10%), mean SALT score 22.3%^§^
			Age- and sex-matched healthy controls (50)	24.48 (6.30)	20 (40)	–
deQueiroz et al. (2022) (27)	Brazil	Case–control	Patients with AA (7)	44.2 (14.90)	7 (100)	NR
			Unmatched controls with other skin conditions (33)	38.8 (16.00)	37 (100)	–
Gao et al. (2022) (28)	China	Case–control	Patients with AA (672)	31.28 (14.42)	276 (41.08)	NR
			Age- and sex-matched healthy controls (580)	30.89 (13.00)	238 (41.03)	–
Goksin (2022) (29)	Turkey	Descriptive	Patients with AA (218)	27.8 (12.30)	84 (38.5)	AU 7 (3.2%), AT 1 (0.5%)
Lim et al. (2022) (30)	USA	Descriptive	Patients with pediatric AA (96)	9 (4.40)	61 (64)	NR
Oner and Akdeniz (2022) (31)	Turkey	Descriptive	Patients with AA (99)	26.1 (12.3)	25 (25.3)	NR
Tran et al. (2022) (16)	USA	Case–control*	Patients with AA (417)	45.70 (NR)	561 (61.60)	NR
			Age-, sex-, and race-matched patients (3127)	49.40	3,685 (74.50)	–
Abedini et al. (2021) (32)	Iran	Case–control	Patients with AA (50)	32.48 (12.61)	23 (46)	Ophiasis 6 (12%), AT 9 (18%), AU 18 (36%)
			Age, sex, and BMI-matched healthy controls (50)	32.26 (12.32)	23 (46)	–
Alamoudi et al. (2021) (33)	Saudi Arabia	Descriptive	Patients with AA (177)	28.37 (12.68)	92 (52)	AU 16 (9%), AT 23 (7%)
Conic et al. (2021) (34)	USA	Descriptive	Patients with AA (77)	37.2^‡^ (NR)	54 (70.10)	NR
Lizarondo et al. (2021) (35)	Philippines	Case–control	Patients with AA (29)	31.48 (10.82)	19 (65.5)	SALT class; S1 = 20 (68.97%), S2 = 5 (17.24%), S3 = 2 (6.90%), S4 = 2 (6.90%), mean SALT score 25.24%^§^
			Age-, sex-, and sun exposure per day-matched healthy controls (29)	31.86 (10.51)	19 (65.5)	–
Conic et al. (2020) (36)	USA	Case–control*	Patients with pediatric AA (3510)	NR	1940 (55.3)	NR
			Unmatched pediatric controls without AA (8310710)	NR	4,018,940 (48.4)	–
Zhao et al. (2020) (14)	China	Case–control	Patients with AA (443)	41.26 (14.10)	279 (62.98)	NR
			Age-, sex-, and season-matched healthy controls (2070)	41.76 (11.25)	1,006 (48.60)	–
Conic et al. (2019) (37)	USA	Descriptive	Patients with AA (18)	71.83 (6.34)	15 (83.3)	NR
El-Ghareeb (2019) (38)	Egypt	Case–control	Patients with AA (20)	NR	NR	NR
			Age-matched healthy controls (20)	NR	NR	–
Marahatta et al. (2019) (39)	Nepal	Case–control	Patients with AA (30)	28.37 (10.07)	14 (48.3)	SALT score = 3.56 ± 3.50%
			Age- and sex-matched healthy controls (30)	30.50 (9.03)	15 (51.7)	–
Namdar and Arikan (2019) (40)	Turkey	Case–control	Patients with AA (60)	31.4 (10.03)	30 (50)	SALT class; S1 = 43 (71.7%), S2 = 17 (28.3%), mean SALT score 19.44%^§^
			Unmatched controls without chronic or dermatological diseases (61)	36.61 (10.08)	27 (44.3)	–
Rehman et al. (2019) (41)	India	Case–control	Patients with AA (135)	26 (12.89)	44 (32.59)	SALT class; S1 = 52 (38.52%), S2 = 35 (25.93%), S3 = 17 (12.59%), S4 = 11 (8.15%), S5 = 7 (5.19%), mean SALT score 38.09%^§^
			Age- and sex-matched healthy controls (135)	26 (13.20)	44 (32.59)	–
Siddappa et al. (2019) – adult AA (42)	India	Case–control	Patients with AA (100)	24.52 (10.06)	28 (28)	SALT class; S1 = 75 (99%), S3 = 1 (1%), mean SALT score 13.14%^§^
			Age- and sex-matched healthy controls (100)	28.96 (11.49)	42 (42)	–
Siddappa et al. (2019) – pediatric AA (43)	India	Case–control	Patients with pediatric AA (30)	11.13 (4.17)	12 (40)	NR
			Age- and sex-matched healthy controls (30)	11.46 (4.41)	12 (40)	–
Daroach et al. (2018) (44)	India	Case–control	Patients with AA (30)	28.97 (9.96)	19 (63.33)	SALT class; S1-2 = 24 (80%), S3-4 = 3 (10%), S5 = 3 (10%), SALT score = 35.8 ± 27.5%
			Age- and sex-matched healthy controls (30)	31.17 (9.43)	14 (46.67)	–
Gade et al. (2018) (45)	India	Case–control	Patients with AA (45)	32.73 (10.43)	31 (68.89)	Median SALT score (%) 4.23 (3.12–6.33)
			Age- and sex-matched healthy controls (45)	33.98 (8.48)	31 (68.89)	–
Karaguzel et al. (2018) (46)	Turkey	Case–control	Patients with pediatric AA (30)	10.5 (2.9)	20 (66)	NR
			Age- and sex-matched healthy pediatric control (30)	10.5 (2.8)	20 (66)	–
Saniee et al. (2018) (15)	Iran	Case–control	Patients with AA (77)	27.38 (11.94)	37 (48.1)	Mean involved area = 43.51 ± 20.25
			Age- and sex-matched normal controls who visited dermatology clinics (112)	29.54 (13.65)	54 (48.2)	–
Unal and Gonulalan (2018) (47)	Turkey	Case–control	Patients with pediatric AA (20)	12.67 (4.16)	6 (30)	SALT class; S1 = 6 (30%), S2 = 9 (45%), S3 = 5 (25%), S4 = 0, S5 = 0, mean SALT score 35.78%^§^
			Unmatched healthy controls (34)	16.54 (0.91)	19 (55.88)	–
Bhat et al. (2017) (48)	India	Case–control	Patients with AA (50)	22.4 (8.6)	NR	SALT class; S1 = 38 (76%), S2 = 12 (24%), mean SALT score 18.38%^§^
			Age- and sex-matched healthy controls randomly recruited from clinic with no history of AA (35)	29.2 (7.6)	NR	–
Conic et al. (2017) (49)	USA	Case–control*	Patients with AA (584)	35.54 (19.28)	400 (68.50)	AT 12 (2.05%), AU 19 (3.25%)
			Age-matched controls with seborrheic dermatitis without hair loss (172)	35.80 (15.56)	126 (73.25)	–
Erpolat et al. (2017) (50)	Turkey	Case–control	Patients with AA (41)	32.8 (7.5)	15 (36.59)	NR
			Unmatched healthy controls (32)	32.7 (7.5)	14 (43.75)	–
Ghafoor and Anwar (2017) (51)	Pakistan	Case–control	Patients with AA (30)	23.77 (8.86)	18 (60)	SALT class; S1 = 4 (13.33%), S2 = 7 (23.33%), S3 = 12 (40%), S4 = 1 (3.33%), S5 = 6 (20%), mean SALT score 57.8%^§^
			Age- and sex-matched healthy volunteers and patients coming to dermatology department for other disorders (30)	24.03 (8.62)	18 (60)	–
Narang et al. (2017) (52)	India	Descriptive	Patients with AA (22)	30.4 (10.8)	10 (45.5)	SALT score ranged 8.4–40
Attawa et al. (2016) (53)	Egypt	Case–control	Patients with AA (23)	26.44 (10.87)	8 (34.8)	SALT class; S1 = 14 (61%), S2 = 3 (13%), S3 + S4 + S5 = 6 (26%), mean SALT score 34.04%^§^
			Unmatched healthy controls (23)	29.39 (8.10)	9 (39.1)	–
Bakry et al. (2016) (54)	Egypt	Case–control	Patients with AA (60)	20.7 (10.85)	24 (40)	Ophiasis 12 (20%), AT/AU 16 (26.7%)
			Age-, sex-, FST-, and BMI-matched healthy controls (60)	23.71 (7.45)	32 (53.3)	–
Darwish et al. (2016) (55)	Egypt	Case–control	Patients with AA (30)	28.67 (10)	17 (56.7)	SALT score: S1 = 10 (33.3%), S2 = 7 (23.3%), S3 = 4 (13.3%), S4 = 3 (10%), S5 = 1 (3.3%), S4 B = 3 (10%), S5 B = 2 (6.7%), mean SALT score 39.64%^§^
			Age- and sex-matched healthy controls (20)	24.8 (6)	10 (50)	–
Fattah and Darwish (2015) (56)	Egypt	Case–control	Patients with AA (30)	26.8 (6.9)	12 (40)	SALT class; S3 = 15 (50%), S4 = 3 (10%), S5 = 12 (40%), B0 = 27 (90%), B2 = 3 (10%), mean SALT score 79.45%^§^
			Age-, sex-, FST-, approximate daily amount of vitamin D intake-, occupation (indoor or outdoor)-, and time of blood sampling- matched healthy controls (30)	25.1 (6.9)	12 (40)	–
Ogrum et al. (2015) (57)	Turkey	Case–control	Patients with AA (40)	31.23 (7.34)	21 (52.5)	SALT class; S1 = 35 (87.5%), S2 = 3 (7.5%), S3 = 2 (5%), mean SALT score 16.79%^§^
			Age-, sex-, and FST-matched healthy controls (40)	30.58 (7.19)	21 (52.5)	–
Cerman et al. (2014) (13)	Turkey	Case–control	Patients with AA (86)	32.21 (9.60)	30 (42)	SALT class; S1 = 41 (83%), S2 = 15 (17%), SALT 14.41 ± 9.92%
			Age- and sex-matched healthy controls (58)	32.55 (9.78)	24 (41.38)	–
Mahamid et al. (2014) (58)	Israel	Case–control	Patients with AA (23)	24.2 (12.3)	9 (39.13)	Extensive 5 (21.74%)
			Age- and sex-matched controls without AA (20)	27 (11.26)	7 (35)	–
D’Ovidio et al. (2013) (59)	Italy	Case–control	Patients with AA (70)	27.79 (9.12)	33 (47.1)	Ophiasis 69 (44%), AT/AU 38 (24.5%)
			Unmatched healthy controls (70)	30.49 (11.06)	26 (37.1)	–
El-Mongy et al. (2013) (60)	Egypt	Case–control	Patients with AA (156)	37.8 (NR)	111 (71.15)	SALT class; S1 = 30 (42.9%), S2 = 12 (17.1%), S3 + S4 + S5 = 28 (40.0%), mean SALT score 44.83%^§^
			Unmatched healthy controls (148)	34.5 (NR)	130 (87.84)	–
Nassiri et al. (2013) (61)^‖^	Iran	Case–control	Patients with AA (28)	27.75 (7.97)	9 (32.14)	SALT (%); 0–24 = 6 (21.4%), 25–49 = 4 (14.3%), 50–74 = 1 (3.6%), and 100 = 17 (60.7%), mean SALT score 70.79%^§^
			Unmatched healthy controls (44)	33.16 (12.52)	28 (63.63)	–
Yilmaz et al. (2012) (62)	Turkey	Case–control	Patients with AA (42)	31.1 (8.2)	28 (66.67)	SALT class; S1 = 30 (71.43%), S2 = 6 (14.29%), S3 = 3 (7.14%), S4 = 2 (4.76%), S5 = 1 (2.38%), mean SALT score 25.13%^§^
			Unmatched healthy controls (42)	29.3 (7.4)	29 (69.05)	–

AA, alopecia areata; BMI, body mass index; FST, Fitzpatrick skin type; NR, not reported; SALT, Severity of Alopecia Tool; USA, United States. * Multicenter studies. ^‡^ Median age. ‖ Vitamin D deficiency defined as < 10 ng/mL. § calculated mean SALT score.

**Table 2:** Characteristics of the included studies involving patients with androgenetic alopecia.

Author, year	Country	Study design	Group (case/control)	Mean age (SD)	Female (%)	Severity of alopecia
Wang et al. (2024) (86)	China	Case–control	Patients with MAGA (40)	27.3 (5.30)	0	NR
			Age- and gender-matched healthy controls (45)	28.3 (4.20)	0	–
Losoya-Jaquez et al. (2024) (25)	Mexico	Descriptive	Patients with pediatric AGA (13^†^)	16.08 (1.30)	42 (21)	NR
Wu et al. (2023) (85)	China	Case–control	Patients with MAGA (80)	36.28 (10.49)	0	Mild 36 (45%), moderate alopecia 37 (46.3%), severe alopecia 7 (0.09%)
			Age-, gender- and BMI-matched healthy controls (60)	36.28 (10.98)	0	–
Vandana et al. (2023) (84)	India	Descriptive	Patients with FPHL (24)	28.9 (NR)	24 (100)	NR
Okhovat et al. (2023) (83)	USA	Descriptive	Patients with FPHL (54^†^)	50.04 (16.40)	54 (100)	NR
Hailat et al. (2023) (82)	Pakistan	Case–control	Patients with FPHL (72)	28.6 (2.40)	72 (100)	NR
			Sex-matched healthy controls (72)	NR	72 (100)	–
Arasu et al. (2022) (70)	Australia	Descriptive	Patients with FPHL (100)	51 (NR)	100 (100)	NR
deQueiroz et al. (2022) (27)	Brazil	Case–control	Patients with FPHL (37)	54.8 (15.00)	37 (100)	NR
			Unmatched controls with other skin conditions (33)	38.8 (16.00)	37 (100)	–
Krysiak et al. (2022) (71)	Poland	Case–control	Patients with MAGA (72)	37 (6.00)	0	NR
			Age-, blood pressure-, BMI-, insulin sensitivity-, and plasma lipids-matched controls without hair loss (75)	38 (6.00)	0	–
Oner and Akdeniz (2022) (31)	Turkey	Descriptive	Patients with AGA (101)	25.6 (7.3)	25 (24.8)	NR
Tran et al. (2022) (16)	USA	Case–control*	Patients with AGA (404)	NR	NR	NR
			Age-, sex-, and race-matched patients (3127)	49.40	3,685 (74.50)	–
Conic et al. (2021) (34)	USA	Descriptive	Patients with AGA (73)	53.2^‡^ (NR)	65 (89)	NR
Danane et al. (2021) (72)	India	Descriptive	Patients with MAGA (50)	24 (NR)	0	NR
El-Tahlawy et al. (2021) (73)	Egypt	Case–control	Patients with MAGA (30)	NR	0	NR
			Age- and sex-matched healthy controls (30)	NR	0	–
Jasim et al. (2021) (74)	Iraq	Case–control	Patients with FPHL (50)	NR	50 (100)	NR
			Unmatched healthy controls (50)	NR	50 (100)	–
Kerkemeyer et al. (2021) (75)	Australia	Descriptive	Patients with MAGA (31^†^)	28.7 (NR)	0	Sinclair grade; 2.0 = 15 (17.6%), 2.5 = 6 (7.1%), 3.0 = 40 (47.6%), 3.5 = 0 (0.0%), 4.0 = 18 (21.4%), 4.5 = 2 (2.4%), and 5.0 = 3 (3.6%)
Sanke et al. (2020) (76)	India	Case–control	Patients with MAGA (50)	21.17 (3.66)	0	Hamilton-Norwood grade; III = 14 (25%), IV = 19 (33%), V = 20 (35%), and grade VI = 4 (7%)
			Age-, sex-, socioeconomic status, and outdoor exposure-matched healthy controls who attended dermatology department (50)	NR	0	–
Zhao et al. (2020) (14)	China	Case–control	Patients with FPHL (657)	32.59 (10.51)	657 (100)	NR
			Patients with MAGA (777)	29.89 (7.02)	0	NR
			Age-, sex-, and season-matched healthy controls (2070)	41.76 (11.25)	1,006 (48.60)	–
Conic et al. (2019) (37)	USA	Descriptive	Patients with FPHL (27)	70.26 (4.99)	27 (100)	NR
Kondrakhina et al. (2019) (77)	Russia	Case–control	Patients with MAGA (50)	26.2 (5.3)	0	NR
			Age- and origin-matched healthy controls (25)	NR	NR	–
Sarac and Koca (2018) (78)	Turkey	Case–control	Patients with AGA (58)	30.3 (8.8)	28 (48.28)	NR
			Unmatched healthy controls (58)	28.5 (10.1)	47 (81.03)	–
Banihashemi et al. (2016) (79)	Iran	Case–control	Patients with FPHL (45)	29.11 (7.31)	45 (100)	NR
			Age-, sex-, hours spent under sunlight per day-, and BMI-matched healthy controls (45)	28.82 (7.11)	45 (100)	–
Moneib et al. (2014) (80)	Egypt	Case–control	Patients with FPHL (60)	28.67 (10)	60 (100)	NR
			Age-, sex-, FPT-, socioeconomic status-, outdoor exposure- matched heatlhy controls (60)	24.8 (6)	60 (100)	–
Rasheed et al. (2013) (81)	Egypt	Case–control	Patients with FPHL (38)	NR	38 (100)	
			Age-, sex-, and FST- matched healthy female controls (40)	30.8 (8.56)	40 (100)	–

AGA, androgenetic alopecia; BMI, body mass index; FPHL, female pattern hair loss; FST, Fitzpatrick skin type; MAGA, male androgenetic alopecia; NR, not reported; USA, United States. * Multicenter studies. ^†^ Number of patients with vitamin D results. ^‡^ Median age.

**Table 3:** Characteristics of the included studies involving patients with telogen effluvium.

Author, year	Country	Study design	Group (case/control)	Mean age (SD)	Female (%)
Vandana et al. (2023) (84)	India	Descriptive	Patients with TE (76)	24.40 (NR)	76 (100)
Arslan et al. (2023) (96)	Turkey	Descriptive	Patients with TE (58^†^)	27.54 (9.42)	840 (86.3)
Chen et al. (2022) (24)	USA	Descriptive	Patients with pediatric TE (68^†^)	12.3 (NR)	67 (88)
deQueiroz et al. (2022) (27)	Brazil	Case–control	Patients with TE (17)	42.8 (14.55)	17 (100)
			Unmatched controls with other skin conditions (33)	38.8 (16.00)	37 (100)
Oner and Akdeniz (2022) (31)	Turkey	Descriptive	Patients with TE (160)	27.7 (8.8)	156 (97.5)
Yorulmaz et al. (2022) (87)	Turkey	Descriptive	Patients with TE (1688^†^)	26^‡^ (NR)	2,794 (92.30)
Alizadeh et al. (2021) (88)	Iran	Case–control	Patients with TE (83)	35^‡^ (NR)	83 (100)
			Age- and sex-matched healthy controls (83)	35^‡^ (NR)	83 (100)
Conic et al. (2021) (34)	USA	Descriptive	Patients with TE (121)	46.9^‡^ (NR)	120 (99.2)
Naser et al. (2021) (89)	Baghdad	Case–control	Patients with TE (60)	32.6 (6.47)	60 (100)
			Age- and sex-matched healthy controls (60)	41.3 (4.59)	60 (100)
Mohammad et al. (2020) (90)	Iran	Case–control	Patients with TE (50)	NR	50 (100)
			Age- and sex-matched healthy controls who referred to dermatology clinic for cosmetic procedures other than hair loss (50)	NR	50 (100)
Sokmen (2020) (91)	Turkey	Descriptive	Patients with TE (151)	29^‡^ (NR)	151 (100)
Conic et al. (2019) (37)	USA	Descriptive	Patients with TE (70)	71.07 (5.35)	68 (97.1)
Cifcia (2018) (92)	Turkey	Case–control	Patients with TE (155)	30.7 (9.80)	149 (96.13)
			Age- and sex-matched healthy controls who visited other clinics for health checkup (168)	30.76 (8.80)	155 (92.26)
Sarac and Koca (2018) (78)	Turkey	Case–control	Patients with TE (71)	26.6 (8.4)	65 (91.55)
			Unmatched healthy controls (58)	28.5 (10.1)	47 (81.03)
Gurel et al. (2017) (93)	Turkey	Case–control	Patients with TE (80)	26.41 (6.93)	80 (100)
			Age- and sex-matched controls without hair loss (80)	25.79 (7.41)	80 (100)
Cheung et al. (2016) (94)	USA	Descriptive	Patients with TE (115^†^)	NR	110 (26.63)
Rasheed et al. (2013) (81)	Egypt	Case–control	Patients with TE (42)	NR	42 (100)
			Age-, sex-, and FST- matched healthy female controls (40)	30.8 (8.56)	40 (100)
Karadag et al. (2011) (95)	Turkey	Case–control	Patients with TE (63)	29.1 (11.9)	63 (100)
			Sex-matched controls without AA (50)	28.4 (9.4)	50 (100)

FST, Fitzpatrick skin type; TE, telogen effluvium; USA, United States. ^†^ Number of patients with vitamin D results. ^‡^ Median age.

**Table 4:** Characteristics of the included studies involving patients with scarring alopecia.

Author, year	Country	Study design	Group (case/control)	Mean age (SD)	Female (%)	Severity of alopecia
Leung et al. (2023) (100)	USA	Case–control	Patients with CCCA (53)	51.3 (9.60)	53 (100)	NR
			Age- and sex-matched healthy controls (212)	50.3 (9.50)	212 (100)	–
Gharaei Nejad et al. (2023) (98)	Iran	Descriptive	Patients with LPP (60)	43.60 (10.17)	44 (73.3)	NR
Arasu et al. (2022) (70)	Australia	Descriptive	Patients with FFA (100)	63 (NR)	100 (100)	NR
Collins et al. (2022) (99)	USA	Descriptive	Patients with CCCA (27)	NR	NR	NR
Conic et al. (2021) (34)	USA	Descriptive	Patients with LPP (58)	56.6^‡^ (NR)	55 (94.8)	NR
			Patients with CCCA (29)	55.2^‡^ (NR)	29 (100)	NR
Conic et al. (2019) (37)	USA	Descriptive	Patients with LPP (37)	69.24 (3.11)	37 (100)	NR
			Patients with FFA (7)	68.86 (3.24)	7 (100)	NR
			Patients with CCCA (4)	67.25 (1.26)	4 (100)	NR
Brankov et al. (2018) (97)	USA	Case–control	Patients with LPP (334)	54.77 (12.83)	311 (93.1)	NR
			Age- and race-matched controls with seborrheic dermatitis without hair loss (78)	52.19 (15.37)	62 (79.5)	–

CCCA, central centrifugal cicatricial alopecia; FFA, frontal fibrosing alopecia; LPP, lichen planopilaris; USA, United States. ^‡^ Median age.

### 3.2 Alopecia areata

Patients with AA were found to have a pooled prevalence of VDD of 51.94% (95% confidence interval: 41.54–62.25%, *I^2^* = 97.48%, *p* < 0.01), a pooled OR VDD of 2.84 (1.89–4.26, *I^2^* = 84.29%, *p* < 0.01), a pooled UMD of −8.20 ng/mL (−10.28 – −6.12 ng/mL, *I^2^* = 74.25%, *p* < 0.01), and a pooled CC of vitamin D level and a SALT score of −0.42 (−0.59 – −0.25, *I^2^* = 85.00%, *p* < 0.01), indicating a significantly higher likelihood of VDD in AA patients compared to controls.

**Figure 2:**
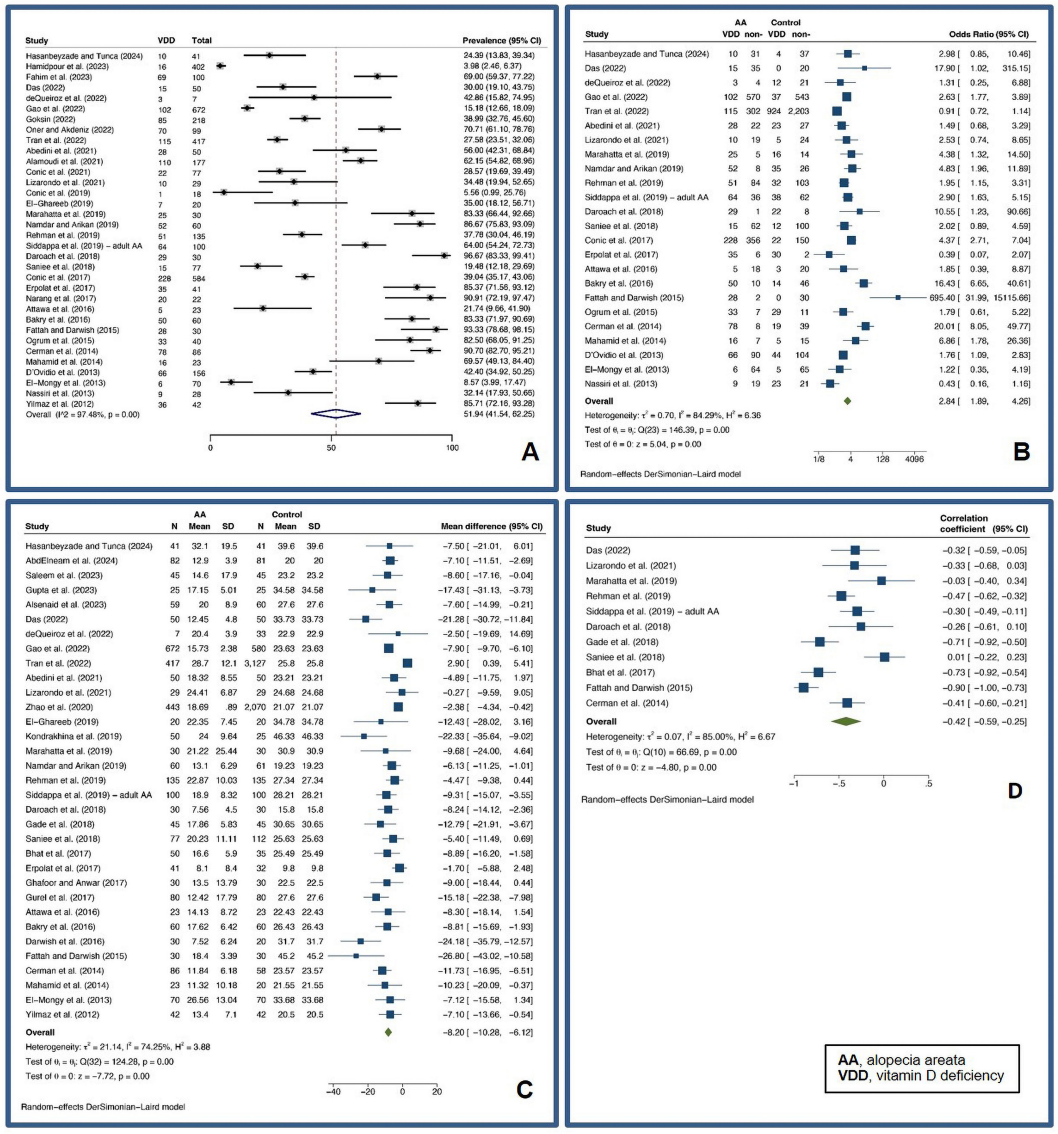
Forest plots for the pooled prevalence of vitamin D deficiency **(A)**, pooled odds ratio of vitamin D deficiency **(B)**, pooled unstandardized mean difference of vitamin D levels **(C)**, and pooled correlation coefficient of vitamin D level and Severity of Alopecia Tool score **(D)** in adult alopecia areata.

Meta-regression analysis revealed that disease duration and relapse may account for heterogeneity in pooled OR analyses, while the female gender may account for heterogeneity in pooled UMD analyses. Subsequent subgroup analyses revealed that a disease duration of 12 months or more had a pooled OR of 2 (1.04–3.83, *I^2^* = 73.87%, *p* < 0.01), whereas a disease duration of less than 12 months had a pooled OR of 11.53 (5.55–23.96, *I^2^* = 46.31%, *p* = 0.13). The pooled OR for cohorts with relapse AA of 50% or more was 8.19 (1.92–35.01, *I^2^* = 71.52%, *p* = 0.03), while the pooled OR for cohorts with relapse AA of less than 50% was 3.62 (1.74–7.55, *I^2^* = 76.96%, *p* < 0.01), which was statistically significant (*p* < 0.01). Cohorts with less than 50% female had a pooled UMD of −8.44 ng/mL (−10.49 – −6.39 ng/mL, *I^2^* = 46.78%, *p* = 0.01), whereas cohorts with more than 50% female had a pooled UMD of −6.94 ng/mL (−10.78–3.11 ng/mL, *I^2^* = 81.01%, *p* < 0.01). Figure 2 demonstrates forest plots for the pooled prevalence of VDD (Figure 2A), pooled odds ratio of VDD (Figure 2B), pooled UMD of vitamin D levels (Figure 2C), and pooled CC of vitamin D level and SALT score (Figure 2D) in adult AA. Supplementary Figure S1 shows subgroup analyses based on disease duration, proportion of relapsed AA, and proportion of females in adult AA.

### 3.3 Pediatric alopecia areata

**Figure 3:**
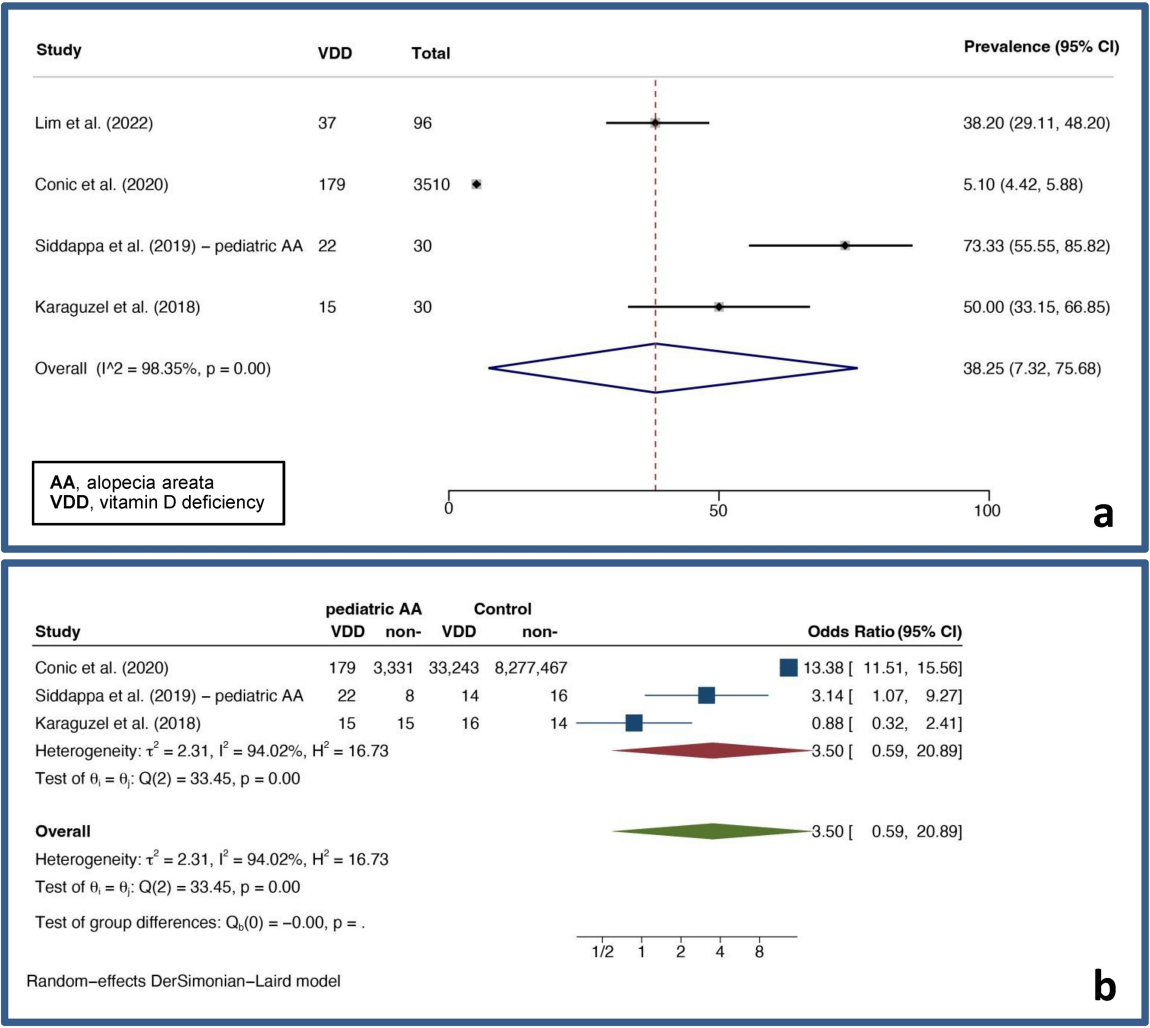
Forest plots for the pooled prevalence of vitamin D deficiency **(A)** and pooled odds ratio of vitamin D deficiency **(B)** in pediatric alopecia areata.

A pooled VDD prevalence and a pooled VDD OR of 38.25% (7.32–75.68%, *I^2^* = 98.35%, *p* < 0.01) and 3.50 (0.59–20.89, *I^2^* = 94.02%, *p* < 0.01) were found, respectively. Figure 3 displays forest plots for the pooled prevalence (Figure 3A) and pooled odds ratio of VDD (Figure 3B) in pediatric AA.

### 3.4 Androgenetic alopecia

**Figure 4:**
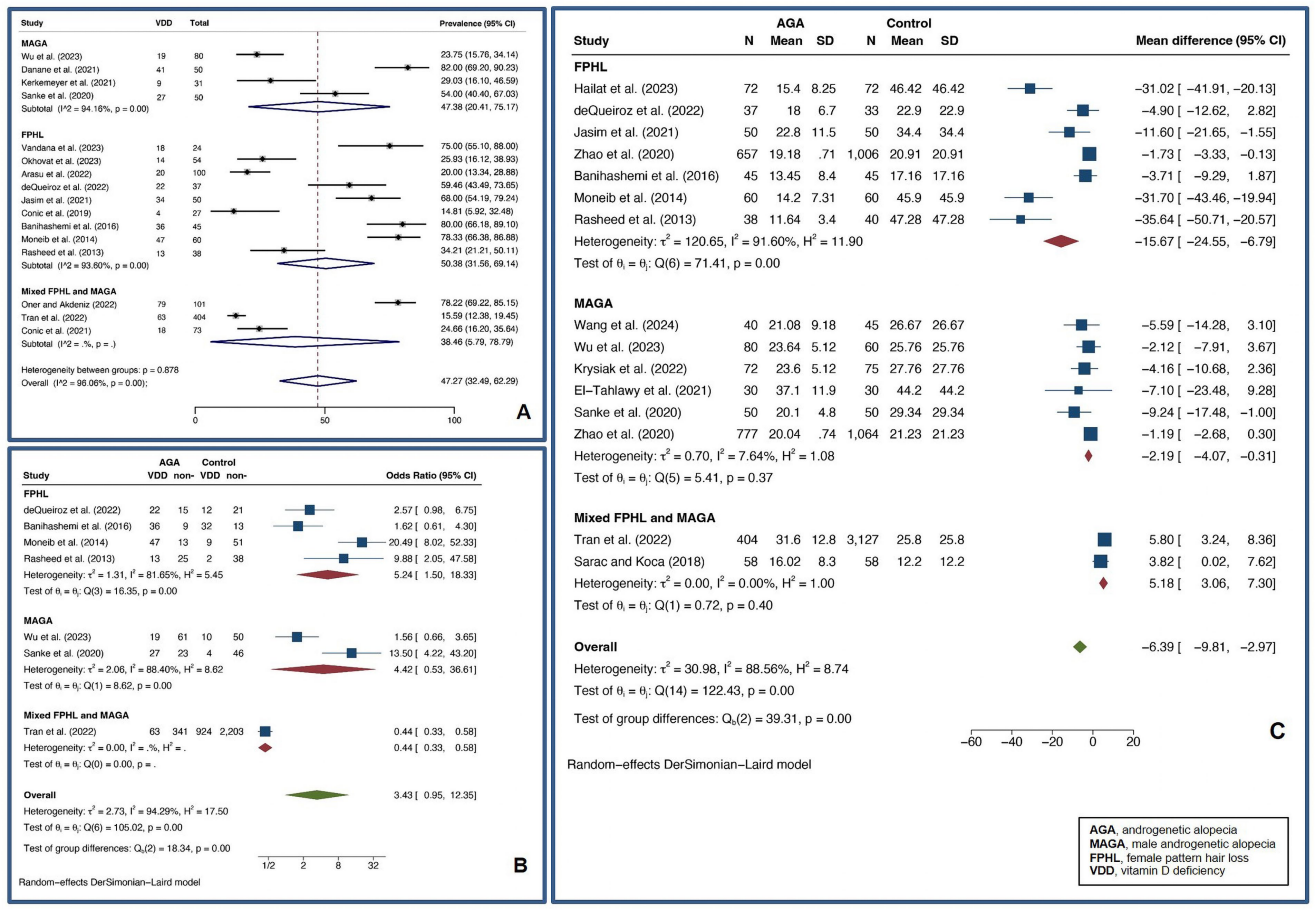
Forest plots for the pooled prevalence of vitamin D deficiency **(A)**, pooled odds ratio of vitamin D deficiency **(B)**, and pooled unstandardized mean difference of vitamin D levels **(C)** in androgenetic alopecia.

A pooled VDD prevalence of 47.27% (32.49–62.29%, *I^2^* = 96.06%, *p* < 0.01) was found for AGA, while a pooled VDD OR of 3.43 (0.95–12.35, *I^2^* = 94.29%, *p* < 0.01) and a pooled UMD of vitamin D levels of −6.39 ng/mL (−9.81 – −2.97 ng/mL, *I^2^* = 88.56%, *p* < 0.01) were found for AGA compared to controls. For FPHL, a pooled VDD prevalence of 50.38% (31.56–69.14%, *I^2^* = 93.60%, *p* < 0.01) was found. Also, a pooled VDD OR of 5.24 (1.50–18.33, *I^2^* = 81.65%, *p* < 0.01) and a pooled UMD of vitamin D levels of −15.67 ng/mL (−24.55 – −6.79 ng/mL, *I^2^* = 91.60%, *p* < 0.01) were found compared to controls, showing a strong association between VDD and FPHL. For MAGA, a pooled VDD prevalence of 47.38% (20.41–75.17%, *I^2^* = 94.16%, *p* < 0.01) was found. For MAGA, a pooled VDD OR of 4.42 (0.53–36.61, *I^2^* = 88.40%, *p* < 0.01), and a pooled UMD of vitamin D levels of −2.19 ng/mL (−4.07 – −0.31 ng/mL, *I^2^* = 7.64%, *p* < 0.37) were found compared to controls. Figure 4 depicts forest plots for the pooled prevalence of VDD (Figure 4A), pooled odds ratio of VDD (Figure 4B), and pooled UMD of vitamin D levels (Figure 4C) in AGA.

### 3.5 Telogen effluvium

**Figure 5:**
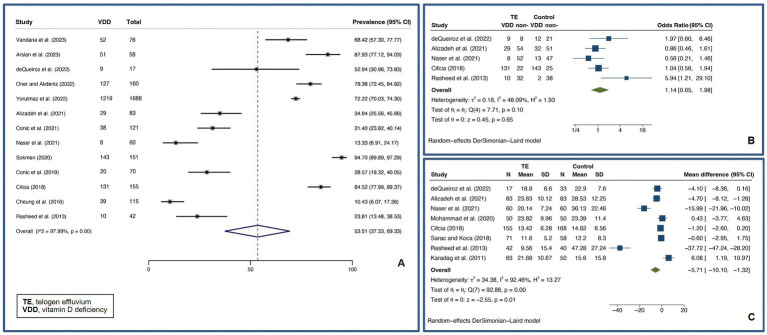
Forest plots for the pooled prevalence of vitamin D deficiency **(A)**, pooled odds ratio of vitamin D deficiency **(B)**, and pooled unstandardized mean difference of vitamin D levels **(C)** in telogen effluvium.

A pooled VDD prevalence of 53.51% (37.33–69.33%, *I^2^* = 97.99%, *p* < 0.01) was found for TE patients. A pooled VDD OR of 1.14 (0.65–1.98, *I^2^* = 48.09%, *p* = 0.10) and a pooled UMD of vitamin D levels of −5.71 ng/mL (−10.10 – −1.32 ng/mL, *I^2^* = 92.46%, *p* < 0.01) were found compared to controls. Figure 5 shows forest plots for the pooled prevalence of VDD (Figure 5A), pooled odds ratio of VDD (Figure 5B), and pooled UMD of vitamin D levels (Figure 5C) in TE.

### 3.6 Primary scarring alopecia

**Figure 6:**
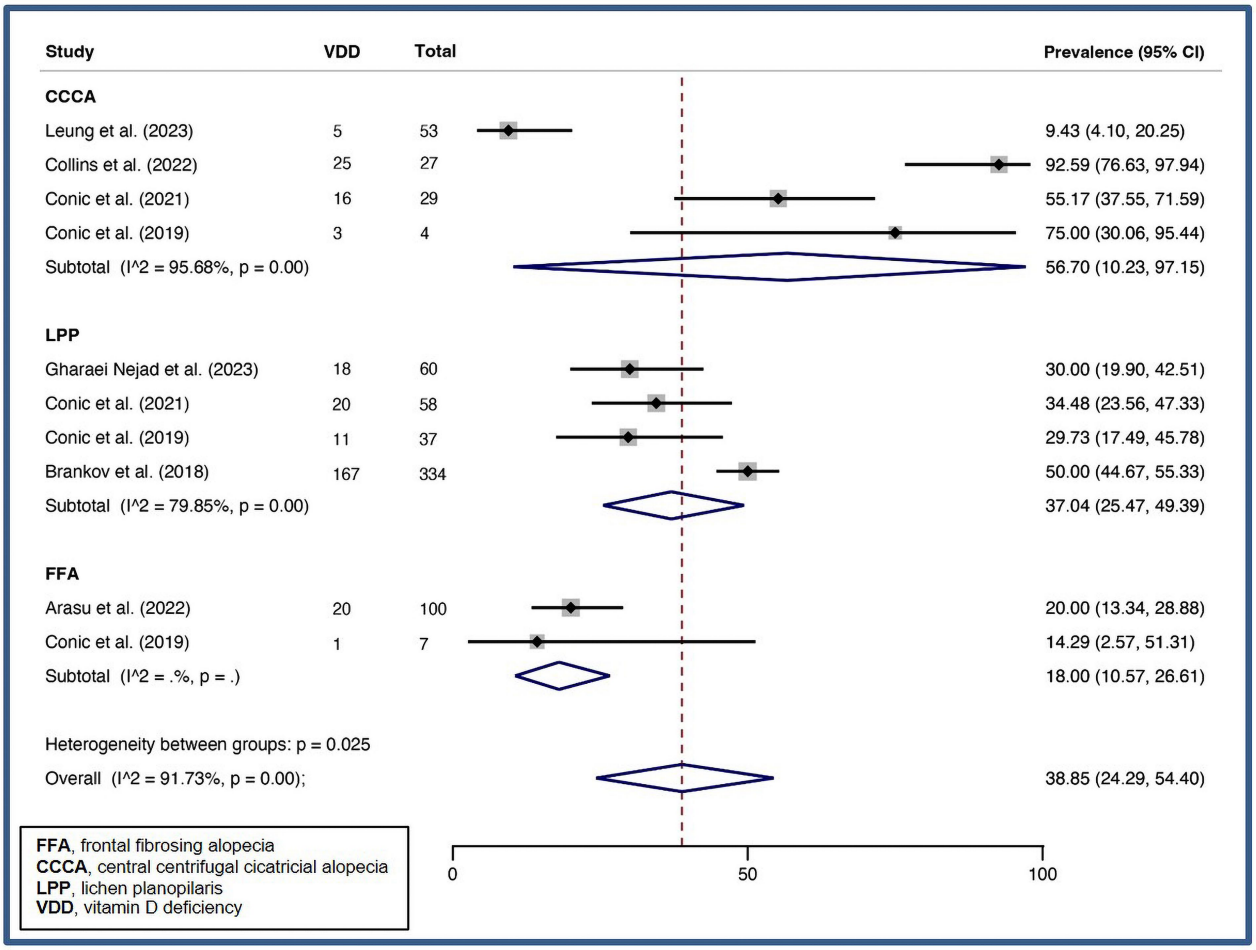
Forest plot for the pooled prevalence of vitamin D deficiency in primary lymphocytic scarring alopecia.

A pooled VDD prevalence of 38.85% (24.29–54.40%, *I^2^* = 91.73%, *p* < 0.01) was found for PCA. Subgroup analysis of specific PCA disorders was performed, and pooled VDD prevalences of 18.00% (10.57–26.61%), 56.70% (10.23–97.15%), and 37.04% (25.47–49.39%) were found for FFA, CCCA, and LPP, respectively. Figure 6 illustrates forest plot for pooled prevalence of VDD in PCA.

### 3.7 Country of research origin subgroup analysis

Pooled VDD prevalences of AA, AGA, and TE in eastern countries of 56.70% (43.04–69.87%, *I^2^* = 97.88%, *p* < 0.01), 64.31% (48.41–78.81%, *I^2^* = 92.10%, *p* < 0.01), and 64.42% (49.17–78.33%, *I^2^* = 97.10%, *p* < 0.01), were found, respectively. Whereas pooled VDD prevalences of 31.36% (23.51–39.74%, *I^2^* = 82.73%, *p* < 0.01), 25.54% (16.80–35.34%, *I^2^* = 82.88%, *p* < 0.01), and 27.56% (13.59–44.07%, *I^2^* = 88.11%, *p* < 0.01) were found for AA, AGA, and TE in western countries.

Pooled VDD ORs of AA, AGA, and TE in eastern countries of 3.18 (2.04–4.97, *I^2^* = 75.74%, *p* < 0.01), 5.65 (1.75–18.18, *I^2^* = 79.96%, *p* < 0.01), and 1.04 (0.56–1.94, *I^2^* = 53.62%, *p* = 0.09) were found, respectively. While pooled VDD ORs of 1.78 (0.77–4.16, *I^2^* = 91.62%, *p* < 0.01), 1.00 (0.18–5.59, *I^2^* = 91.52%, *p* < 0.01), and 1.97 (0.60–6.46) were found for AA, AGA, and TE in western countries.

Pooled UMDs of vitamin D levels of AA, AGA, and TE in eastern countries of −8.24 ng/mL (−10.02 – −6.46 ng/mL, *I^2^* = 58.11%, *p* < 0.01), −8.13 ng/mL (−11.97 – −4.29 ng/mL, *I^2^* = 87.64%, *p* < 0.01), and − 6.07 ng/mL (−11.02 – −1.13 ng/mL, *I^2^* = 93.47%, *p* < 0.01) were found, respectively. However, pooled UMDs of vitamin D levels of −6.67 ng/mL (−23.12–9.77 ng/mL, *I^2^* = 85.29%, *p* < 0.01), and − 0.52 ng/mL (−8.58–7.54 ng/mL, *I^2^* = 84.55%, *p* < 0.01) were found for AA and AGA in western countries. Table 5 summarizes subgroup analyses based on the country of research origin.

**Table 5:** Pre-planned subgroup analyses based on country of research origin, age group, and alopecia severity.

	Alopecia areata			Androgenetic alopecia			Telogen effluvium		
Subgroup	No. of studies	Prevalence	I^2^ (%)/*p*-value of Q test	No. of studies	Prevalence	I^2^ (%)/*p*-value of Q test	No. of studies	Prevalence	I^2^ (%)/*p*-value of Q test
Overall	34	51.94% (41.54–62.25%)	97.48/<0.01	16	47.27% (32.49–62.29%)	96.06/<0.01	13	53.51% (37.33–69.33%)	97.99/<0.01
Country									
Eastern	28	56.70% (43.04–69.87%)	97.88/<0.01	9	64.31% (48.41–78.81%)	92.10/<0.01	9	64.42% (49.17–78.33%)	97.10/<0.01
Western	6	31.36% (23.51–39.74%)	82.73/<0.01	7	25.54% (16.80–35.34%)	82.88/<0.01	4	27.56% (13.59–44.07%)	88.11/<0.01
Age									
Mean age 18–25 years	3	47.01% (1.32–96.78%)	–	3	70.69% (51.32–86.96%)	–	0	–	–
Mean age 25–60 years	29	54.67% (44.56–64.60%)	96.79/<0.01	11	42.07% (25.49–59.60%)	96.42/<0.01	9	56.00% (38.63–72.67%)	97.70/<0.01
Mean age > 60 years	1	5.56% (0.99–25.76%)	–	1	14.81% (5.92–32.48%)	–	1	28.57% (19.32–40.05%)	–
Unspecified	1	35.00% (18.12–56.71%)	–	1	68.00% (54.19–79.24%)	–	1	10.43% (6.07–17.36%)	–
Severity									
Severe cohorts	9	44.36% (19.70–70.54%)	98.01/<0.01	–	–	–	–	–	–
Non-severe cohorts	13	63.71% (47.43–78.58%)	95.25/<0.01						
Unspecified	12	44.64% (31.88–57.75%)	96.90/<0.01						

Subgroup	No. of studies	Odds ratio	I^2^ (%)/*p*-value of Q test	No. of studies	Odds ratio	I^2^ (%)/*p*-value of Q test	No. of studies	Odds ratio	I^2^ (%)/*p*-value of Q test
Overall	24	2.84 (1.89–4.26)	84.29/<0.01	7	3.43 (0.95–12.35)	95.16/<0.01	5	1.14 (0.65–1.98)	48.09/0.10
Country									
Eastern	20	3.18 (2.04–4.97)	75.74/<0.01	5	5.65 (1.75–18.18)	79.96/<0.01	4	1.04 (0.56–1.94)	53.62/0.09
Western	4	1.78 (0.77–4.16)	91.62/<0.01	2	1.00 (0.18–5.59)	91.52/<0.01	1	1.97 (0.60–6.46)	–
Age									
Mean age 18–25 years	2	6.65 (1.22–36.35)	90.06/<0.01	1	13.50 (4.22–43.21)	–	0	–	–
Mean age 25–60 years	22	2.57 (1.70–3.88)	82.36/<0.01	6	2.73 (0.74–10.15)	93.99/<0.01	5	1.14 (0.65–1.98)	48.09/0.10
Severity									
Severe cohorts	8	3.29 (1.30–8.34)	85.53/<0.01	–	–	–	–	–	–
Non-severe cohorts	11	3.58 (2.20–5.82)	60.92/<0.01						
Unspecified	5	1.62 (0.71–3.71)	91.62/<0.01						

Subgroup	No. of studies	Mean difference	I^2^ (%)/*p*-value of Q test	No. of studies	Mean difference	I^2^ (%)/*p*-value of Q test	No. of studies	Mean difference	I^2^ (%)/*p*-value of Q test
Overall	33	−8.20 (−10.28 – −6.12)	74.25/<0.01	15	−6.39 (−9.81 – −2.97)	88.56/<0.01	8	−5.71 (−10.10 – −1.32)	92.46/<0.01
Country									
Eastern	30	−8.24 (−10.02 – −6.46)	58.11/<0.01	12	−8.13 (−11.97 – −4.29)	87.64/<0.01	7	−6.07 (−11.02 – −1.13)	93.47/<0.01
Western	3	−6.67 (−23.12–9.77)	85.29/<0.01	3	−0.52 (−8.58–7.54)	84.55/<0.01	1	−4.10 (−8.36–0.16)	–
Age									
Mean age 18–25 years	5	−8.98 (−12.23 – −5.73)	0.00/1.00	1	−9.24 (−11.28 – −7.20)	–	0	–	–
Mean age 25–60 years	27	−8.07 (−10.44 – −5.70)	78.12/<0.01	12	−5.81 (−9.49 – −2.13)	90.34/<0.01	7	−6.74 (−11.69 – −1.80)	93.44/<0.01
Mean age > 60 years	0	–	–	0	–	–	0	–	–
Unspecified	1	−12.43 (−18.47 – −6.39)	–	2	−10.37 (−18.94 – −1.80)	88.56%/0.65	1	0.43 (−3.77–4.63)	–
Severity									
Severe cohorts	8	−10.65 (−15.23 – −6.07)	44.83/0.08	–	–	–	–	–	–
Non-severe cohorts	17	−8.17 (−9.97 – −6.37)	15.12/0.28						
Unspecified	8	−5.92 (−10.36 – −1.47)	89.79/<0.01						

### 3.8 Age group subgroup analysis

AA and AGA cohorts with mean ages 18–25 years were found to have pooled VDD prevalences of 47.01% (1.32–96.78%) and 70.69% (51.32–86.96%), respectively, while AA and AGA cohorts with mean ages 25–60 years were found to have pooled VDD prevalences of 54.67% (44.56–64.60%, *I^2^* = 96.79, *p* < 0.01) and 42.07% (25.49–59.60%, *I^2^* = 96.42, *p* < 0.01), respectively.

AA cohorts with mean age 18–25 years were found to have a pooled VDD OR of 6.65 (1.22–36.35, *I^2^* = 90.06, *p* < 0.01), while AA cohorts with mean age 25–60 years were found to have a pooled VDD OR of 2.57 (1.70–3.88, *I^2^* = 82.36%, *p* < 0.01). AA cohorts with mean age 18–25 years were found to have a pooled UMD of vitamin D levels of −8.98 ng/mL (−12.23 – −5.73 ng/mL, *I^2^* = 0.00, *p* = 1.00); however, AA cohorts with mean age 25–60 years were found to have a pooled UMD of vitamin D levels of −8.07 ng/mL (−10.44 – −5.70 ng/mL, *I^2^* = 78.12, *p* < 0.01). Table 5 presents subgroup analyses based on age group.

### 3.9 Alopecia severity subgroup analysis

Severe AA cohorts were found to have a VDD prevalence of 44.36% (19.70–70.54%, *I^2^* = 98.01%, *p* < 0.01), VDD OR of 3.29 (1.30–8.34, *I^2^* = 85.53%, *p* < 0.01), a UMD of vitamin D levels of −10.65 ng/mL (−15.23 – −6.39 ng/mL, *I^2^* = 44.83%, *p* = 0.08), while non-severe AA cohorts were found to have a VDD prevalence of 63.71% (47.43–78.58%, *I^2^* = 95.25%, *p* < 0.01), VDD OR of 3.58 (2.20–5.82, *I^2^* = 60.92%, *p* < 0.01), and a UMD of vitamin D levels of −8.17 ng/mL (−9.97 – −6.37 ng/mL, *I^2^* = 15.12%, *p* = 0.28). Due to insufficient information, subgroup analyses based on other alopecia disorders were not conducted. Table 5 shows subgroup analyses based on alopecia severity.

### 3.10 Quality assessment

Supplementary Table S3 provides a summary of the quality assessment scores for comparative and descriptive studies included in the review. The average quality assessment score was 7.22 (range: 5–9), with 59 high-quality and 22 fair-quality studies. Sensitivity analyses based on study quality were performed to assess the robustness of the findings. The results were consistent with the primary analyses, suggesting that potential biases did not significantly influence the pooled estimates (Supplementary Document 2).

### 3.11 Publication bias

Some funnel plots were slightly asymmetric when assessing publication bias for each primary analysis (Supplementary Figure S2). As a result, Egger’s tests were conducted, and it was discovered that some analyses exhibited possible asymmetry; consequently, we performed additional contour-enhanced funnel plots. We discovered that the asymmetry in the VDD OR analyses for AA and AGA, UMD of vitamin D levels analysis in TE and AA, and CC of vitamin D level and a SALT score were likely due to heterogeneity. In the UMD of vitamin D levels analysis in AGA, however, publication bias is highly likely. Funnel plots and contour-enhanced funnel plots are shown in Supplementary Figure S2.

## 4 Discussion

Our analysis revealed that VDD was prevalent among patients with various alopecia disorders, including AA, FPHL, MAGA, TE, and PCA. Statistically significant associations were observed in AA and FPHL patients, who demonstrated a higher likelihood of VDD and lower vitamin D levels compared to controls, although MAGA and TE patients also exhibited lower vitamin D levels compared to controls. Geographical factors exerted an influence, as the prevalence of VDD and the reduction in vitamin D levels were more pronounced in studies conducted in Eastern countries than in Western countries. Furthermore, younger patients aged 18 to 25 years exhibited a higher prevalence of VDD and more severe reductions in vitamin D levels compared to older patients. The study also determined that patients with severe AA exhibited greater reductions in vitamin D levels compared to controls, though both severe and non-severe AA patients had comparable VDD prevalence. Overall, the findings suggest a significant relationship between vitamin D deficiency and certain types of alopecia, particularly AA and FPHL, underscoring the necessity for further research into the role of vitamin D in these conditions.

The role of vitamin D as an immunomodulator is particularly relevant in AA, in which autoimmune mechanisms are hypothesized to play a pivotal role. Vitamin D modulates the activity of cytotoxic T cells, regulatory T cells, and dendritic cells, all of which are involved in AA pathogenesis. Insufficient vitamin D levels may contribute to dysregulation of the immune response in AA, potentially resulting in an autoimmune attack on hair follicles (101).

Previously, there have been a few meta-analyses on VDD and AA. Similar to previous studies, we found that VDD is prevalent among AA, and compared to controls, AA had significantly higher odds of VDD and significantly lower vitamin D levels (102–104). In addition to updating the previous systematic review, we also pooled CC of AA disease severity and found a significant negative correlation between SALT scores and vitamin D levels, and severe AA was found to have a greater reduction of vitamin D level compared to control (*vs* non-severe AA). However, both severe and non-severe AA cohorts have similar odds of VDD. Currently, no cohort study has investigated the causal relationship between AA and VDD. Therefore, it is unknown whether VDD initiates AA pathogenesis or exacerbates AA conditions or whether AA causes VDD. A longitudinal study that investigates the connection between AA and VDD would provide evidence and strengthen the recommendation to screen AA patients for VDD.

A significantly stronger association between VDD and AA was observed in cohorts with AA patients with a disease duration of less than 1 year, suggesting that vitamin D status may play a crucial role in the early stages of AA development. Additionally, studies with age group of 18-to-25 years showed a higher risk of VDD, indicating that young adults with AA might be especially vulnerable to vitamin D deficiency. Interestingly, cohorts with a higher proportion of relapsed AA were found to have a higher risk of VDD, which aligns with the understanding of AA as an autoimmune disease (7). The relapsed state may represent compromised immune regulation, potentially exacerbated by low vitamin D levels (3, 7, 105–107). In contrast, cohorts with a lower proportion of females had lower vitamin D levels, hinting at possible gender-specific differences in vitamin D metabolism or AA pathogenesis. These findings underscore the complex interplay between vitamin D status and AA, highlighting the need for further research to elucidate the specific role of age, gender, and disease duration in this relationship. Such investigations could provide valuable insights into the pathophysiology of AA and potentially inform more targeted therapeutic approaches.

As for pediatric AA, current evidence indicates that the prevalence and likelihood of VDD are lower than in the adult population, with non-statistically significant odds of having VDD compared to pediatric controls. Current evidence is relatively limited, and additional well-controlled studies are required to clarify the significance of VDD in pediatric AA.

To our knowledge, this is the first systematic review and meta-analysis to investigate the association between VDD and non-AA hair loss disorders, specifically AGA, TE, and scarring alopecia. Both TE and AGA were found to have a VDD prevalence of approximately 50% and significantly lower vitamin D levels than controls but did not have an increased risk of VDD compared to controls. However, the likelihood of VDD in FPHL patients is statistically significantly higher than in controls. Vitamin D levels in FPHL cohorts are significantly lower than in controls compared to AGA in general. Although MAGA data is limited, we hypothesize that gender plays a significant role in VDD and AGA (108, 109).

Our study found that in Eastern countries, the prevalence of vitamin D deficiency, the likelihood of VDD, and the degree of lower vitamin D levels among AA, AGA, and TE were significantly higher than in Western nations. European Caucasians have a lower prevalence of VDD than non-white individuals (110). In addition to differences in skin pigmentation, the use of sunscreen and latitude are also significant factors that could cause VDD by reducing vitamin D synthesis (111–113). Melanin in darker skin tones can interfere with vitamin D synthesis, while widespread sunscreen use and higher latitudes with less direct sunlight can reduce vitamin D production (114). Moreover, because these factors are rarely matched but could significantly influence the results, additional studies matching sunscreen use and FST are required.

Due to the limited number of case–control studies, the relationship between VDD and scarring alopecia remains poorly understood. However, our analysis suggests that the prevalence of VDD may vary among different types of PCA. Based on available data, certain PCA diseases may have a higher VDD prevalence than others. For instance, CCCA has a VDD prevalence of 57%, whereas FFA has a VDD prevalence of 18%. However, the higher prevalence of VDD among CCCA patients may not be due to the disease itself but rather their skin pigmentation, as it almost exclusively affects females with FST V-VI, which are known to be associated with a higher risk of VDD due to reduced vitamin D synthesis in darker skin (111, 115). To establish a more definitive association between scarring alopecia and VDD, and to better understand the role of confounding factors such as skin type, additional well-designed controlled studies are required.

The results of our study should be interpreted with caution due to the highly heterogeneous study population and setting of the included studies, as well as the fact that serum vitamin D levels can be affected by a variety of factors, such as geographic characteristics, ethnicity, and skin tone (111). Subgroup analyses were conducted to explore potential sources of heterogeneity; however, for most alopecia types, significant sources of heterogeneity were not identified, suggesting that residual heterogeneity may be attributed to unmeasured variables or other contextual factors not captured in the included studies. Future research should focus on more detailed reporting and examination of factors contributing to heterogeneity. Also, publication bias exists in the analyses of the pooled UMD in vitamin D levels between AGA and controls, which necessitates caution when interpreting our results.

The study has several strengths and limitations. One of the strengths is its comprehensive approach, utilizing a systematic review and meta-analysis to pool data from a wide range of studies. The study also follows rigorous methodological standards, including the registration of the protocol in PROSPERO, adherence to PRISMA guidelines, and the use of validated tools such as the NOS for quality assessment, which contributes to the robustness of the conclusions. However, the study has some limitations. The heterogeneity of the included studies suggests considerable variability in study populations, settings, and methodologies, which could affect the reliability of the pooled estimates. Moreover, the study’s reliance on observational data limits the ability to infer causality. Finally, the limited number of studies on scarring alopecia and pediatric populations restricts the generalizability of the findings to these subgroups.

## 5 Conclusion

Even though VDD is prevalent among alopecia patients, the likelihood of VDD and decreased vitamin D levels compared to the control population was statistically significant only in adult AA and FPHL. Adult AA disease severity was found to be significantly negatively correlated with vitamin D levels, with severe AA cohorts showing a higher reduction of vitamin D levels compared to controls; however, severe and non-severe AA cohorts appear to have comparable VDD prevalence and VDD likelihood compared to controls. Cohorts with less than one year of AA duration and a higher proportion of relapsed AA were found to have a higher risk of VDD, while cohorts with a lower proportion of females had lower vitamin D levels. Studies conducted in Eastern nations appear to report a higher VDD prevalence, VDD likelihood, and vitamin D reduction than studies conducted in Western nations. Evidence is still lacking for MAGA, scarring alopecia, and pediatric AA, highlighting the need for further research in these areas. It is important to acknowledge the limitations of this study, including the heterogeneity of the included studies. Given these results, clinicians should consider routine screening for VDD in patients with severe AA or FPHL, particularly in Eastern countries or in patients with recent onset or relapsed AA. Early detection and potential correction of vitamin D deficiency could play a role in managing the severity and progression of alopecia. Future studies should focus on addressing the gaps in our understanding of the role of vitamin D in alopecia, particularly MAGA, scarring alopecia, and pediatric AA, as well as investigating the potential benefits of vitamin D supplementation in alopecia management.

## Data Availability

The original contributions presented in the study are included in the article/Supplementary material, further inquiries can be directed to the corresponding author.

## References

[1] Mora JR Iwata M von Andrian UH. Vitamin effects on the immune system: vitamins a and D take Centre stage. Nat Rev Immunol. (2008) 8:685–98. doi: 10.1038/nri2378 19172691 PMC2906676

[2] Ersoy-Evans S . Commentary: Vitamin D and autoimmunity: is there an association? J Am Acad Dermatol. (2010) 62:942–4. doi: 10.1016/j.jaad.2010.02.009 20466171

[3] Hewison M . An update on Vitamin D and human immunity. Clin Endocrinol. (2012) 76:315–25. doi: 10.1111/j.1365-2265.2011.04261.x 21995874

[4] Arnson Y Amital H Shoenfeld Y. Vitamin D and autoimmunity: new Aetiological and therapeutic considerations. Ann Rheum Dis. (2007) 66:1137–42. doi: 10.1136/ard.2007.069831, PMID: 17557889 PMC1955167

[5] Doche I Romiti R Hordinsky MK Valente NS. “Normal-appearing” scalp areas are also affected in lichen Planopilaris and frontal Fibrosing alopecia: an observational histopathologic study of 40 patients. Exp Dermatol. (2020) 29:278–81. doi: 10.1111/exd.13834, PMID: 30403306

[6] Dankers W Colin EM van Hamburg JP Lubberts E. Vitamin D in autoimmunity: molecular mechanisms and therapeutic potential. Front Immunol. (2017) 7:7. doi: 10.3389/fimmu.2016.00697, PMID: 28163705 PMC5247472

[7] Suchonwanit P Kositkuljorn C Pomsoong C. Alopecia Areata: an autoimmune disease of multiple players. Immunotargets Ther. (2021) 10:299–312. doi: 10.2147/itt.S266409 34350136 PMC8328385

[8] Harries MJ Paus R. The pathogenesis of primary Cicatricial Alopecias. Am J Pathol. (2010) 177:2152–62. doi: 10.2353/ajpath.2010.100454, PMID: 20889564 PMC2966773

[9] Harnchoowong S Suchonwanit P. Ppar-Γ agonists and their role in primary Cicatricial alopecia. PPAR Res. (2017) 2017:2501248. doi: 10.1155/2017/2501248, PMID: 29333153 PMC5733188

[10] Malloy PJ Feldman D. The role of Vitamin D receptor mutations in the development of alopecia. Mol Cell Endocrinol. (2011) 347:90–6. doi: 10.1016/j.mce.2011.05.045, PMID: 21693169 PMC3196847

[11] Bikle DD Vitamin D. Metabolism and function in the skin. Mol Cell Endocrinol. (2011) 347:80–9. doi: 10.1016/j.mce.2011.05.017 21664236 PMC3188673

[12] Solomon JD Heitzer MD Liu TT Beumer JH Parise RA Normolle DP . Vdr activity is differentially affected by Hic-5 in prostate Cancer and stromal cells. Mol Cancer Res. (2014) 12:1166–80. doi: 10.1158/1541-7786.Mcr-13-0395 24825850 PMC4134986

[13] Aksu Cerman A Sarikaya Solak S Kivanc AI. Vitamin D deficiency in alopecia Areata. Br J Dermatol. (2014) 170:1299–304. doi: 10.1111/bjd.12980 24655364

[14] Zhao J Sheng Y Dai C Qi S Hu R Rui W . Serum 25 Hydroxyvitamin D levels in alopecia Areata, female pattern hair loss, and male androgenetic alopecia in a Chinese population. J Cosmet Dermatol. (2020) 19:3115–21. doi: 10.1111/jocd.13396 32275116

[15] Saniee S Zare AG Radmehr A. Evaluating the serum zinc and Vitamin D levels in alopecia Areata. Iranian J Dermatol. (2018) 21:77–80. doi: 10.22034/ijd.2018.98360

[16] Tran PT Chen A Yi L Goh C. Vitamin D levels in alopecia Areata and other Alopecias: a retrospective case- control study at a single institution. Int J Trichology. (2022) 14:175–7. doi: 10.4103/ijt.ijt_131_20, PMID: 36404883 PMC9674059

[17] Moher D Liberati A Tetzlaff J Altman DG. Preferred reporting items for systematic reviews and Meta-analyses: the Prisma statement. BMJ. (2009) 339:b2535. doi: 10.1136/bmj.b2535, PMID: 19622551 PMC2714657

[18] Holick MF . Sunlight and Vitamin D for bone health and prevention of autoimmune diseases, cancers, and cardiovascular disease. Am J Clin Nutr. (2004) 80:1678s–88s. doi: 10.1093/ajcn/80.6.1678S 15585788

[19] Wells G Shea B O'Connell D Peterson J Welch V Losos M . The Newcastle–Ottawa scale (Nos) for assessing the quality of non-randomized studies in Meta-analysis. (2000). Available at: http://www.ohri.ca/programs/clinical_epidemiology/oxford.htm (Accessed 2024 September 26).

[20] Nyaga VN Arbyn M Aerts M. Metaprop: a Stata command to perform Meta-analysis of binomial data. Arch Public Health. (2014) 72:39. doi: 10.1186/2049-3258-72-39, PMID: 25810908 PMC4373114

[21] Deeks JJ Altman DG Bradburn MJ. Statistical methods for examining heterogeneity and combining results from several studies in Meta-analysis. Syst Rev Health Care. (2001):285–312. doi: 10.1002/9780470693926.ch15

[22] Thompson SG . Why sources of heterogeneity in Meta-analysis should be investigated. BMJ. (1994) 309:1351–5. doi: 10.1136/bmj.309.6965.1351, PMID: 7866085 PMC2541868

[23] Sterne JA Egger M Smith GD. Systematic reviews in health care: investigating and dealing with publication and other biases in Meta-analysis. BMJ. (2001) 323:101–5. doi: 10.1136/bmj.323.7304.101, PMID: 11451790 PMC1120714

[24] Chen V Strazzulla L Asbeck SM Bellodi SF. Etiology, management, and outcomes of pediatric Telogen effluvium: a single-center study in the United States. Pediatr Dermatol. (2022) 40:120–4. doi: 10.1111/pde.15154, PMID: 36263718

[25] Losoya-Jaquez MR Lopez Yañez-Blanco A Armendariz-Barragan Y Aguilar-Figueroa NG Rudnicka L Sanchez-Dueñas LE. Androgenetic alopecia in children and adolescents: from Trichoscopy to therapy. Skin Appendage Disord. (2024) 10:123–8. doi: 10.1159/000534844, PMID: 38572189 PMC10987064

[26] Das AK . A hospital-based observational evaluation of serum 25-Hydroxy Vitamin D levels in alopecia Areata of scalp. Int J Toxicol Pharmacol Res. (2022) 12:241–7.

[27] de Queiroz M Vaske TM Boza JC. Serum ferritin and Vitamin D levels in women with non-scarring alopecia. J Cosmet Dermatol. (2022) 21:2688–90. doi: 10.1111/jocd.14472, PMID: 34564937

[28] Gao Y Huo S Sun M Zhang C Wang J Gao J . Evaluation of several immune and inflammatory indicators and their association with alopecia Areata. J Cosmet Dermatol. (2022) 21:2995–3001. doi: 10.1111/jocd.14504, PMID: 34591347

[29] Goksin S . Retrospective evaluation of clinical profile and comorbidities in patients with alopecia Areata. North Clin Istanb. (2022) 9:451–8. doi: 10.14744/nci.2022.78790, PMID: 36447582 PMC9677058

[30] Lim RK Castelo-Soccio L Putterman E Qureshi AA Cho E. Predictors of Vitamin D insufficiency in children and adolescents with alopecia Areata. Cureus. (2022) 14:e22934. doi: 10.7759/cureus.22934, PMID: 35399430 PMC8986346

[31] Öner Ü Akdeniz N. Nonscarring scalp alopecia: which laboratory analysis should we perform on whom? Turkish J Med Sci. (2022) 52:188–94. doi: 10.3906/sag-2106-28, PMID: 34688244 PMC10734875

[32] Abedini R Shakiba S Ghandi N Yazdaniamjad F Haddadi NS Nasimi M. Study of Vitamin D deficiency in patients with alopecia Areata attending a dermatology Center in Iran. Iranian J Dermatol. (2021) 24:97–101. doi: 10.22034/ijd.2021.132456

[33] Alamoudi SM Marghalani SM Alajmi RS Aljefri YE Alafif AF. Association between Vitamin D and zinc levels with alopecia Areata phenotypes at a tertiary care center. Cureus. (2021) 13:e14738. doi: 10.7759/cureus.14738 34079683 PMC8162299

[34] Conic RRZ Piliang M Bergfeld W Atanaskova-Mesinkovska N. Vitamin D status in scarring and nonscarring alopecia. J Am Acad Dermatol. (2021) 85:478–80. doi: 10.1016/j.jaad.2018.04.032, PMID: 29689324 PMC6196125

[35] Lizarondo FPJ Gervasio MKR Chamberlin CVS Gnilo CMS Silva CY. Determination of serum 25-Hydroxyvitamin D levels in patients with alopecia Areata and their comparison with levels in healthy controls: a cross-sectional study. JAAD Int. (2021) 5:78–84. doi: 10.1016/j.jdin.2021.07.008, PMID: 34622224 PMC8484040

[36] Conic RR Tamashunas NL Damiani G Fabbrocini G Cantelli M Bergfeld WF. Comorbidities in pediatric alopecia Areata. J Europ Acad Dermatol Venereol. (2020) 34:2898–901. doi: 10.1111/jdv.16727 32531131

[37] Conic RRZ Juhasz M Rambhia P Damiani G Atanaskova-Mesinkovska N Piliang M . Characterizing hair loss in the elderly: an observational study of 163 patients. J Eur Acad Dermatol Venereol. (2019) 33:e226–8. doi: 10.1111/jdv.15462, PMID: 30828889

[38] El-Ghareeb M . Deficient and/or insufficient serum 25 Hydroxy Vitamin D in patients with alopecia Areata: is it a fact or a fiction? Egyptian J Dermatol Venerol. (2019) 39:21. doi: 10.4103/ejdv.ejdv_25_18

[39] Marahatta S Agrawal S Khan S. Study on serum Vitamin D in alopecia Areata patients. J Nepal Health Res Counc. (2019) 17:21–5. doi: 10.33314/jnhrc.v17i01.1475 31110371

[40] Namdar ND Arikan I. The relationship between Vitamin D levels and the quality of life in patients with alopecia Areata and vitiligo. Turk Osteoporoz Dergisi. (2019) 25:35–9. doi: 10.4274/TOD.GALENOS.2019.19327

[41] Rehman F Dogra N Wani M. Serum Vitamin D levels and alopecia Areata- a hospital based case-control study from North-India. Int J Trichology. (2019) 11:49–57. doi: 10.4103/ijt.ijt_3_19, PMID: 31007473 PMC6463459

[42] Siddappa H Kumar YHK Vivekananda N. Evaluation of Association of Vitamin D in alopecia Areata: a case-control study of 100 patients in a tertiary rural Hospital of Southern India. Indian Dermatol Online J. (2019) 10:45–9. doi: 10.4103/idoj.IDOJ_84_18, PMID: 30775298 PMC6362755

[43] Siddappa H Yadalla H Neladimmanahally V. Evaluation of Vitamin D in pediatric alopecia Areata: a case–control study of thirty patients in a tertiary care hospital. Indian J Paediatr Dermatol. (2019) 20:32. doi: 10.4103/ijpd.IJPD_83_18

[44] Daroach M Narang T Saikia UN Sachdeva N Sendhil KM. Correlation of Vitamin D and Vitamin D receptor expression in patients with alopecia Areata: a clinical paradigm. Int J Dermatol. (2018) 57:217–22. doi: 10.1111/ijd.13851, PMID: 29243839

[45] Gade VKV Mony A Munisamy M Chandrashekar L Rajappa M. An investigation of Vitamin D status in alopecia Areata. Clin Exp Med. (2018) 18:577–84. doi: 10.1007/s10238-018-0511-8, PMID: 29869122

[46] Karaguzel G Sakarya NP Bahadir S Beyhun E Yaman S. Vitamin D Status and the Effect of Oral Vitamin D Treatment in Children with Alopecia Areata. J Steroids Horm Sci. (2018) 9:189. doi: 10.4172/2157-7536.1000189

[47] Unal M Gonulalan G. Serum Vitamin D level is related to disease severity in pediatric alopecia Areata. J Cosmet Dermatol. (2018) 17:101–4. doi: 10.1111/jocd.12352, PMID: 28447433

[48] Bhat YJ Latif I Malik R Hassan I Sheikh G Lone KS . Vitamin D Level in Alopecia Areata. Indian J Dermatol. (2017) 62:407–10. doi: 10.4103/ijd.IJD_677_16, PMID: 28794553 PMC5527723

[49] Conic RZ Miller R Piliang M Bergfeld W Atanaskova MN. Comorbidities in patients with alopecia Areata. J Am Acad Dermatol. (2017) 76:755–7. doi: 10.1016/j.jaad.2016.12.007 28325393

[50] Erpolat S Sarifakioglu E Ayyildiz A. 25-Hydroxyvitamin D status in patients with alopecia Areata. Postepy Dermatol Alergol. (2017) 34:248–52. doi: 10.5114/ada.2017.67847, PMID: 28670255 PMC5471380

[51] Ghafoor R Anwar MI. Vitamin D deficiency in alopecia Areata. J College Phys Surg Pakistan. (2017) 27:200–2. PMID: 28492146

[52] Narang T Daroach M Kumaran MS. Efficacy and safety of topical Calcipotriol in Management of Alopecia Areata: a pilot study. Dermatol Ther. (2017) 30:e12464. doi: 10.1111/dth.12464, PMID: 28133875

[53] Attawa EMKA Elbalaat W Samy AM. Assessment of Vitamin D level in patients of alopecia Areata. J Clin Investigat Dermatol. (2016) 4:4. doi: 10.13188/2373-1044.1000030

[54] Bakry OA El Farargy SM El Shafiee MK Soliman A. Serum Vitamin D in patients with alopecia Areata. Indian Dermatol Online J. (2016) 7:371–7. doi: 10.4103/2229-5178.190504, PMID: 27730032 PMC5038097

[55] Darwish N Marzok H Gaballah M Abdellatif H. Serum level of Vitamin D in patients with alopecia Areata. Egyptian J Basic Appl Sci. (2016) 4:9–14. doi: 10.1016/j.ejbas.2016.12.001

[56] Fattah NSAA Darwish YW. Assessment of serum 25-Hydroxyvitamin D levels in patients with extensive/recalcitrant alopecia Areata before and after Puva and Nb-Uvb therapy. J Egyp Women's Dermatol Soc. (2015) 12:19–23. doi: 10.1097/01.EWX.0000450679.92939.42

[57] Oʇrum A Boyraz N Toʇral AK Karasati S Ekşioʇlu HM. Evaluation of 25 Hydroxy Vitamin D3 levels in patients with alopecia Areata. Turkderm Deri Hastaliklari ve Frengi Arsivi. (2015) 49:50–3. doi: 10.4274/turkderm.07888

[58] Mahamid M Abu-Elhija O Samamra M Mahamid A Nseir W. Association between Vitamin D levels and alopecia Areata. Israel Med Ass J. (2014) 16:367–70. 25058999

[59] D'Ovidio R Vessio M D'Ovidio FD. Reduced level of 25-Hydroxyvitamin D in chronic/relapsing alopecia Areata. Dermatoendocrinol. (2013) 5:271–3. doi: 10.4161/derm.24411, PMID: 24194967 PMC3772915

[60] El-Mongy NN El-Nabarawy E Hassaan SA Younis ER Shaker O. Serum 25-Hydroxy Vitamin D3 level in Egyptian patients with alopecia Areata. J Egyp Women’s Dermatol Soc. (2013) 10:37–41. doi: 10.1097/01.EWX.0000419612.74665.2b

[61] Nassiri S Saffarian Z Younespour S. Association of Vitamin D Level with alopecia Areata. Iranian J Dermatol. (2013) 16:1–5.

[62] Yilmaz N Serarslan G Gokce C. Vitamin D concentrations are decreased in patients with alopecia Areata. Vitamins & Trace. Elements. (2012) 1:01. doi: 10.4172/2167-0390.1000105

[63] AbdElneam AI Al-Dhubaibi MS Bahaj SS Mohammed GF Alantry AK Atef LM. C-reactive protein as a novel biomarker for Vitamin D deficiency in alopecia Areata. Skin Res Technol. (2024) 30:e13657. doi: 10.1111/srt.13657, PMID: 38528743 PMC10963905

[64] Alsenaid A Al-Dhubaibi MS Alhetheli G AbdElneam AI. Trichoscopy pattern and evaluation of serum Vitamin D status in alopecia Areata. Photodiagn Photodyn Ther. (2023) 42:103510. doi: 10.1016/j.pdpdt.2023.103510, PMID: 36944416

[65] Fahim M Khoso H Hussain A Bakhtiar R. Deficiency in alopecia Areata and responsiveness to Vitamin D analogues: a prospective trial. J Pak Assoc Dermatol. (2023) 33:1242–8.

[66] Gupta SS Mahendra A Gupta S Singla R. Serum Vitamin D levels in alopecia Areata: a case-control study. Iranian J Dermatol. (2023) 26:1–5. doi: 10.22034/ijd.2023.169888

[67] Hamidpour E Shakoei S Nasimi M Ghandi N. Effects of age and sex on the comorbidities of alopecia Areata: a cross-sectional hospital-based study. Health Sci Rep. (2023) 6:e1444. doi: 10.1002/hsr2.1444, PMID: 37519427 PMC10372299

[68] Hasanbeyzade S Tunca M. Vitamin D levels and their relationship with the type of involvement in alopecia Areata patients. J Pak Assoc Dermatol. (2024) 34:200–7.

[69] Saleem S Erfan M Bacha MF Khan I Jabbar A Akram S. Prevalence of Vitamin-D deficiency among individuals diagnosed with alopecia Areata. Med Forum Month. (2023) 34:66–9. doi: 10.60110/medforum.341216

[70] Arasu A Meah N Eisman S Wall D Sinclair R. Vitamin D status in patients with frontal Fibrosing alopecia: a retrospective study. JAAD Int. (2022) 7:129–30. doi: 10.1016/j.jdin.2022.03.008, PMID: 35497640 PMC9043390

[71] Krysiak R Kowalcze K Okopień B. Impaired metabolic effects of metformin in men with early-onset androgenic alopecia. Pharmacol Rep. (2022) 74:216–28. doi: 10.1007/s43440-021-00347-8, PMID: 34897595 PMC8786753

[72] Danane AMG Agrawal S. Study of Vitamin D levels in men with premature androgenetic alopecia. Paripex Indian J Res. (2021) 10:41–2. doi: 10.36106/paripex/1503642

[73] Tahlawy S Alkhayat M Samhoud E. Serum Vitamin D and serum ferritin levels in male pattern hair loss: is there a role? Fayoum Univ Med J. (2021) 8:1–8. doi: 10.21608/fumj.2021.184699

[74] Jasim KI Khidhair ASMA Hussain GA. Assessment of serum Vit D and serum ferritin in female pattern androgenic alopecia. Indian J Forensic Med Toxicol. (2021) 15:3343–51. doi: 10.37506/ijfmt.v15i3.15818

[75] Kerkemeyer KL de Carvalho LT Jerjen R John J Sinclair RD Pinczewski J . Female pattern hair loss in men: a distinct clinical variant of androgenetic alopecia. J Am Acad Dermatol. (2021) 85:260–2. doi: 10.1016/j.jaad.2020.09.042, PMID: 32950545

[76] Sanke S Samudrala S Yadav A Chander R Goyal R. Study of serum Vitamin D levels in men with premature androgenetic alopecia. Int J Dermatol. (2020) 59:1113–6. doi: 10.1111/ijd.14982, PMID: 32516435

[77] Kondrakhina IN Verbenko DA Zatevalov AM Kubanov AA Deryabin DG. The value of genetic and non-genetic factors in the emergence and in the development of androgenetic alopecia in men: multifactor analysis. Vestnik Rossiiskoi Akademii Meditsinskikh Nauk. (2019) 74:167–75. doi: 10.15690/vramn1141

[78] Saraç GKT . The importance of Vitamin-D in androgenic alopecia and Telogen effluvium. J Clin Med Kazakhstan. (2018) 4:26–9. doi: 10.23950/1812-2892-JCMK-00601

[79] Banihashemi M Nahidi Y Meibodi NT Jarahi L Dolatkhah M. Serum Vitamin D3 level in patients with female pattern hair loss. Int J Trichology. (2016) 8:116–20. doi: 10.4103/0974-7753.188965, PMID: 27625563 PMC5007917

[80] Moneib H Fathy G Ouda A. Possible Association of Female-Pattern Hair Loss with alteration in serum 25-Hydroxyvitamin D levels. Egypt J Dermatol Venerol. (2014) 34:15–20. doi: 10.4103/1110-6530.137254

[81] Rasheed H Mahgoub D Hegazy R El-Komy M Abdel Hay R Hamid MA . Serum ferritin and Vitamin D in female hair loss: do they play a role? Skin Pharmacol Physiol. (2013) 26:101–7. doi: 10.1159/000346698, PMID: 23428658

[82] Hailat R Mughal H Malik T Sagheer A Javed A Memon Q. Possible Association of Female-Pattern Hair Loss with alteration in serum 25-Hydroxyvitamin D levels. Pakistan J Med Health Sci. (2023) 17:623–5. doi: 10.53350/pjmhs2023172623

[83] Okhovat JP Marks DH Manatis-Lornell A Hagigeorges D Senna MM. Utility of laboratory testing in patients with female pattern hair loss. J Am Acad Dermatol. (2023) 88:153–5. doi: 10.1016/j.jaad.2019.07.015, PMID: 31306723

[84] Vandana D Gudi SD Raghuveer C Pattar LY. Biochemical correlates of diffuse non scarring alopecia in women. J Cardiovasc Dis Res. (2023) 14:553–9. doi: 10.48047/jcdr.2023.14.07.56

[85] Wu Y Hui Y Liu F Chen H Liu K Chen Q . The Association of Serum Adipokines, insulin resistance and Vitamin D status in male patients with androgenetic alopecia. Clin Cosmet Investig Dermatol. (2023) 16:419–27. doi: 10.2147/ccid.S396697 PMC9936883 36817642

[86] Wang S Xu S Wang S Fang W Shi W. Risk factors and lipid metabolism characteristics of early-onset male androgenetic alopecia: a pilot study. J Cosmet Dermatol. (2024) 23:3038–44. doi: 10.1111/jocd.16371, PMID: 38738464

[87] Yorulmaz A Hayran Y Ozdemir AK Sen O Genc I Gur Aksoy G . Telogen effluvium in daily practice: patient characteristics, laboratory parameters, and treatment modalities of 3028 patients with Telogen effluvium. J Cosmet Dermatol. (2022) 21:2610–7. doi: 10.1111/jocd.14413 34449961

[88] Alizadeh N Rafiei R Darjani A Eftekhari H Nejad K Rafiei E . Chronic Telogen effluvium in women: role of micronutrients, a case-control study in north of Iran. J Egypt Women's Dermatol Soc. (2021) 18:205–9. doi: 10.4103/jewd.jewd_34_21

[89] Naser RT Fadheel QJ Hafedh AH. The significance of serum ferritin and Vitamin D levels in females patients with chronic Telogen effluvium. Indian J Forensic Med Toxicol. (2021) 15:3992–4000. doi: 10.37506/ijfmt.v15i3.15920

[90] Mohammad AP Baba A Ghassemi M. Comparison between serum levels of Vitamin D and zinc in women with diffuse non-scarring hair loss (Telogen effluvium) and healthy women. Pakistan J Med Health Sci. (2020) 14:1400–4.

[91] Sökmen F . The etiological role of thyroid dysfunction and Vitamin deficiency in patients with Telogen effluvium. Osmangazi J Med. (2020) 42:705–9. doi: 10.20515/otd.775603

[92] Çifcia N . Evaluation of Vitamin D levels in chronic Telogen effluvium patients. Turkiye Klinikleri Dermatoloji. (2018) 28:51–5. doi: 10.5336/dermato.2018-61736

[93] Gürel G Karadöl M Çölgeçen E. The role of ferritin and Vitamin D levels in Telogen effluvium. Turkiye Klinikleri Dermatoloji. (2017) 27:113–6. doi: 10.5336/dermato.2017-56970

[94] Cheung EJ Sink JR English Iii JC. Vitamin and mineral deficiencies in patients with Telogen effluvium: a retrospective cross-sectional study. J Drugs Dermatol. (2016) 15:1235–7. PMID: 27741341

[95] Karadaǧ AS Ertuǧrul DT Tutal E Akin KO. The role of Anemia and Vitamin D levels in acute and chronic Telogen effluvium. Turkish J Med Sci. (2011) 41:827–33. doi: 10.3906/sag-1005-853

[96] Arslan H Gündüz Ö. Micronutrient deficiencies and digital computerized Phototrichogram analysis in Telogen effluvium: a retrospective correlation study in a tertiary medical center. Dermatol Pract Concept. (2023) 13:e2023202. doi: 10.5826/dpc.1303a202, PMID: 37557102 PMC10412014

[97] Brankov N Conic RZ Atanaskova-Mesinkovska N Piliang M Bergfeld WF. Comorbid conditions in lichen Planopilaris: a retrospective data analysis of 334 patients. Int J Women's Dermatol. (2018) 4:180–4. doi: 10.1016/j.ijwd.2018.04.001, PMID: 30175224 PMC6116820

[98] Gharaei Nejad K Ghadarjani R Eftekhari H Sheykholeslami S. Most frequent comorbidities in patients with lichen Planopilaris: a cross-sectional study. Int J Dermatol Venereol. (2023) 6:229–32. doi: 10.1097/JD9.0000000000000306

[99] Collins MS Ali S Wiss IP Senna MM. Increased risk of Vitamin D deficiency and insufficiency in black patients with central centrifugal Cicatricial alopecia. J Am Acad Dermatol. (2022) 87:689–91. doi: 10.1016/j.jaad.2022.02.018, PMID: 35176399

[100] Leung B Lindley L Reisch J Glass DA Ayoade K. Comorbidities in patients with central centrifugal Cicatricial alopecia: a retrospective chart review of 53 patients. J Am Acad Dermatol. (2023) 88:461–3. doi: 10.1016/j.jaad.2022.06.013, PMID: 35716835

[101] Zeng Y Yang S Liu Y Tang Z Zong X Li X . The role of Vd/Vdr signaling pathway in autoimmune skin diseases. Mini Rev Med Chem. (2023) 23:652–61. doi: 10.2174/1389557523666221124123206, PMID: 36424786

[102] Lee S Kim BJ Lee CH Lee WS. Increased prevalence of Vitamin D deficiency in patients with alopecia Areata: a systematic review and Meta-analysis. J Eur Acad Dermatol Venereol. (2018) 32:1214–21. doi: 10.1111/jdv.14987 29633370

[103] Lee S Lee H Lee CH Lee WS. Comorbidities in alopecia Areata: a systematic review and Meta-analysis. J Am Acad Dermatol. (2019) 80:466–77.e16. doi: 10.1016/j.jaad.2018.07.013 30031145

[104] Liu Y Li J Liang G Cheng C Li Y Wu X. Association of Alopecia Areata with Vitamin d and calcium levels: a systematic review and Meta-analysis. Dermatol Ther (Heidelb). (2020) 10:967–83. doi: 10.1007/s13555-020-00433-4, PMID: 32772238 PMC7477029

[105] Chanprapaph K Mahasaksiri T Kositkuljorn C Leerunyakul K Suchonwanit P. Prevalence and risk factors associated with the occurrence of autoimmune diseases in patients with alopecia Areata. J Inflamm Res. (2021) 14:4881–91. doi: 10.2147/jir.S331579 34588794 PMC8473714

[106] Suchonwanit P Kositkuljorn C Mahasaksiri T Leerunyakul K. A comparison of the efficacy and tolerability of three corticosteroid treatment regimens in patients with alopecia Areata. J Dermatolog Treat. (2022) 33:756–61. Epub 2020/05/23. doi: 10.1080/09546634.2020.1773384, PMID: 32441159

[107] Chanprapaph K Pomsoong C Kositkuljorn C Suchonwanit P. Intramuscular Corticosteroid Therapy in the treatment of alopecia Areata: a time-to-event analysis. Drug Des Devel Ther. (2022) 16:107–16. doi: 10.2147/dddt.S342179 PMC8752075 35027820

[108] Suchonwanit P Triyangkulsri K Ploydaeng M Leerunyakul K. Assessing biophysical and physiological profiles of scalp seborrheic dermatitis in the Thai population. Biomed Res Int. (2019) 2019:5128376:1–6. doi: 10.1155/2019/5128376, PMID: 31360714 PMC6644260

[109] Chanprapaph K Sutharaphan T Suchonwanit P. Scalp biophysical characteristics in males with androgenetic alopecia: a comparative study with healthy controls. Clin Interv Aging. (2021) 16:781–7. doi: 10.2147/cia.S310178, PMID: 34007163 PMC8122003

[110] Amrein K Scherkl M Hoffmann M Neuwersch-Sommeregger S Köstenberger M Tmava Berisha A . Vitamin D deficiency 2.0: an update on the current status worldwide. Eur J Clin Nutr. (2020) 74:1498–513. doi: 10.1038/s41430-020-0558-y, PMID: 31959942 PMC7091696

[111] Holick MF Vitamin D. Deficiency. N Engl J Med. (2007) 357:266–81. doi: 10.1056/NEJMra070553 17634462

[112] Mahasaksiri T Kositkuljorn C Anuntrangsee T Suchonwanit P. Application of topical immunotherapy in the treatment of alopecia Areata: a review and update. Drug Des Devel Ther. (2021) 15:1285–98. doi: 10.2147/dddt.S297858, PMID: 33790540 PMC8001176

[113] Rattanakaemakorn P Suchonwanit P. Scalp pruritus: review of the pathogenesis, diagnosis, and management. Biomed Res Int. (2019) 2019:1268430–11. doi: 10.1155/2019/1268430, PMID: 30766878 PMC6350598

[114] Guo J Lovegrove JA Givens DI. A narrative review of the role of foods as dietary sources of Vitamin D of ethnic minority populations with darker skin: the underestimated challenge. Nutrients. (2019) 11:81. doi: 10.3390/nu11010081, PMID: 30609828 PMC6356726

[115] Aguh C McMichael A. Central Centrifugal Cicatricial Alopecia. JAMA. Dermatology. (2020) 156:1036. doi: 10.1001/jamadermatol.2020.1859, PMID: 32745206

