# Supplementary figures and images for: Vitamin D deficiency in non-scarring and scarring alopecias: a systematic review and meta-analysis
Source: Front Nutr. 2024 Oct 2;11:1479337. doi: 10.3389/fnut.2024.1479337 (PMC11479915; doi:10.3389/fnut.2024.1479337)

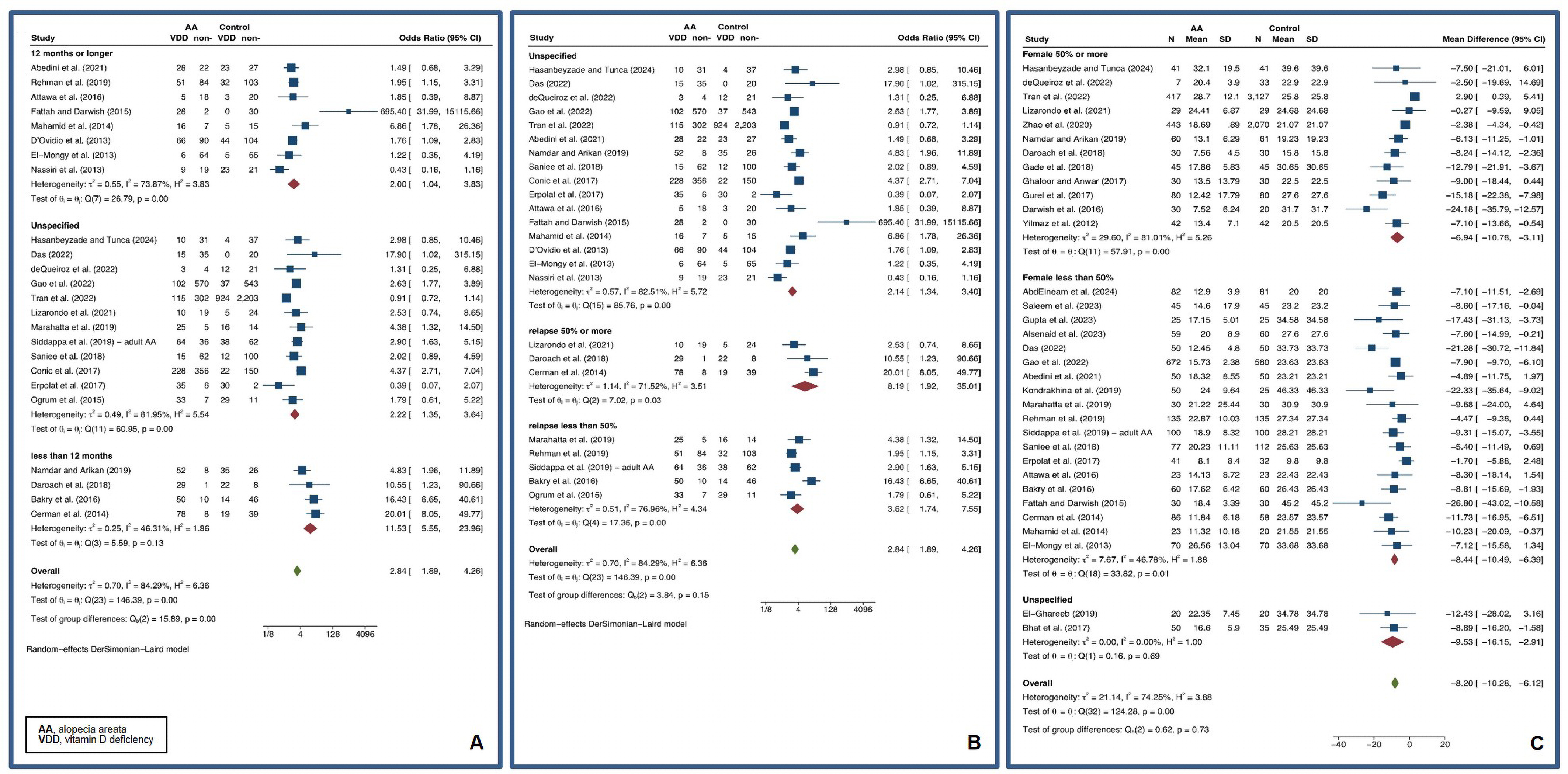

Supplement: Supplementary file 1 [file Image_1.JPEG]

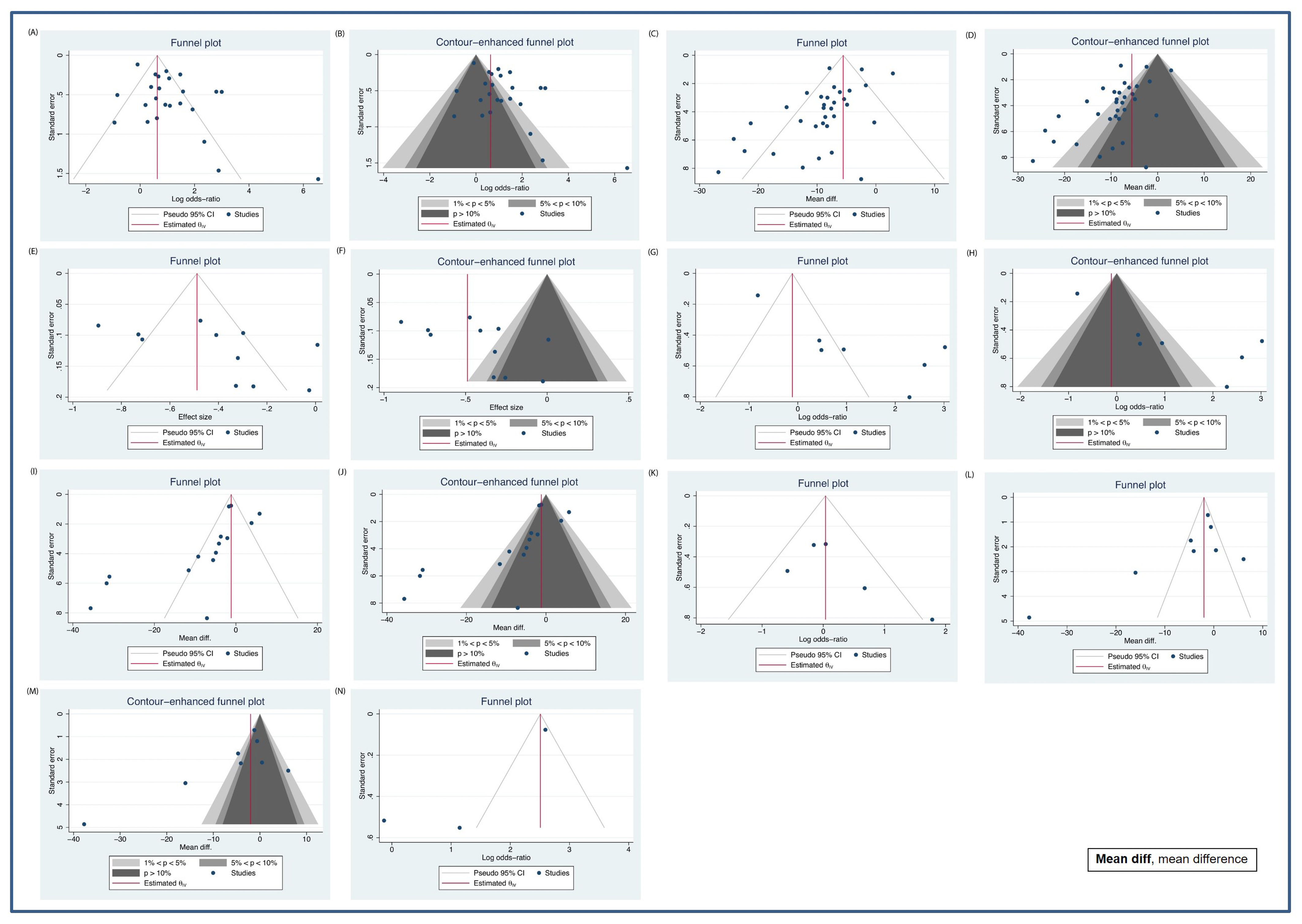

Supplement: Supplementary file 2 [file Image_2.JPEG]
