# Supplementary material for: Vitamin D deficiency in non-scarring and scarring alopecias: a systematic review and meta-analysis
Source: Front Nutr. 2024 Oct 2;11:1479337. doi: 10.3389/fnut.2024.1479337 (PMC11479915; doi:10.3389/fnut.2024.1479337)
Supplement: Supplementary file 3 [file Table_2.docx]

**Supplementary** **Document 2**: Sensitivity analyses

| **Analysis** | **All studies** | **NOS 7 or higher** |
| --- | --- | --- |
| AA prevalence | 51.94% (41.54–62.25%), *I^2^*=97.48%, p<0.01 | 53.68% (37.76–69.24%), *I^2^* = 97.76%, p<0.01 |
| AA OR | 2.84 (1.89–4.26), *I^2^*= 84.29%, p<0.01 | 4.48 (2.75–7.29), *I^2^* = 74%, p<0.01 |
| AA MD | -8.20 (-10.28– -6.12), *I^2^*=74.25%, p<0.01 | -9.60 (-12.05– -7.15), *I^2^* = 65.47%, p<0.01 |
| AGA prevalence | 47.27% (32.49–62.29%), *I^2^*=96.06%, p<0.01 | 43.11% (27.01–59.96%), *I^2^* = 93.72%, p<0.01 |
| AGA OR | 3.43 (0.95–12.35), *I^2^*=94.29%, p<0.01 | 7.85 (2.05–30.06), *I^2^* = 83.65%, p<0.01 |
| AGA MD | -6.39 (-9.81– -2.97), *I^2^*=88.56%, p<0.01 | -6.01 (-9.50– -2.53), *I^2^* = 83.79%, p<0.01 |
| TE prevalence | 53.51% (37.33–69.33%) *I^2^*=97.99%, p<0.01 | 52.15% (34.16–69.86%), *I^2^* = 98.32%, p<0.01 |
| TE OR | 1.14 (0.65–1.98), *I^2^*=48.09%, p=0.10 | 1.04 (0.45–19.43), *I^2^* = 53.62%, p=0.09 |
| TE MD | -5.71 (-10.10– -1.32), *I^2^*=92.46%, p<0.01 | -4.59 (-0.77–0.59), *I^2^* = 85.82%, p<0.01 |
